# Impact of model assumptions on the inference of the evolution of ectomycorrhizal symbiosis in fungi

**DOI:** 10.1038/s41598-022-26514-2

**Published:** 2022-12-21

**Authors:** Sanea Sheikh, Faheema Kalsoom Khan, Mohammad Bahram, Martin Ryberg

**Affiliations:** 1 Program in Systematic Biology, Uppsala University, Norbyvägen 18D, 752 43 Uppsala, Sweden; 2 Section of Terrestrial Ecology, University of Copenhagen, Universitetsparken 15, 2100 Copenhagen, Denmark; 3 Department of Ecology, Swedish University of Agricultural Sciences, Ulls Väg 16, 756 51 Uppsala, Sweden; 4 Institute of Ecology and Earth Sciences, University of Tartu, Vanemuise 46, 51014 Tartu, Estonia

**Keywords:** Evolution, Phylogenetics

## Abstract

Ectomycorrhiza (ECM) is a symbiotic relation between plant and fungi that is essential for nutrient uptake of many stand forming trees. There are two conflicting views about the evolution of ECM in fungi suggesting (1) relatively few transitions to ECM followed by reversals to non-ECM, or (2) many independent origins of ECM and no reversals. In this study, we compare these, and other, hypotheses and test the impact of different models on inference. We assembled a dataset of five marker gene sequences (nuc58, nucLSU, nucSSU, rpb1, and rpb2) and 2,174 fungal taxa covering the three subphyla: Agaricomycotina, Mucoromycotina and Pezizomycotina. The fit of different models, including models with variable rates in clades or through time, to the pattern of ECM fungal taxa was tested in a Bayesian framework, and using AIC and simulations. We find that models implementing variable rates are a better fit than models without rate shift, and that the conclusion about the relative rate between ECM and non-ECM depend largely on whether rate shifts are allowed or not. We conclude that standard constant-rate ancestral state reconstruction models are not adequate for the analysis of the evolution of ECM fungi, and may give contradictory results to more extensive analyses.

## Introduction

The study of trait evolution is an important part of understanding organismal diversity as differences in traits also impact many other dimensions of biodiversity, for example functional diversity. Consequently, ancestral state reconstruction methods have become very popular. Model-based ancestral state reconstruction methods not only offer means to infer the character state at ancestral nodes, but by comparing different models, it also allows to make inferences about the evolutionary process that generates the pattern in diversity amongst taxa. However, in order to understand the evolution of traits with complex histories, we need inclusive phylogenies for power of inference^1^ and models that adequately represent the evolutionary process.

Fungi vary greatly in how they obtain their carbon, and form different functional guilds largely depending on their nutritional mode. As one major fungal guild, ectomycorrhizal (ECM) fungi play important roles in critical ecosystem functions while forming a root associated symbiotic relation with plants^2^. These fungi provide nutrients, water, and other benefits to the plant in return for energy rich carbon compounds^3^. Although not the only type of mycorrhiza, it is the most diverse type among fungi^4^, involving more than 20,000 fungal species in many separate clades^5,6^. On the plant side, ECM fungi form associations with disparate groups^7,8^. While ECM fungi depend on their plant partner in natural conditions^9^, some ECM plants are capable of forming other types of mycorrhizae^8^. ECM symbiosis is especially prevalent among woody plants, and many stand forming trees are dependent on this kind of mycorrhiza^8^. So even if only 2% of the plant species form ECM, they represent about 60% of the tree stems on earth^10^, and consequently have a global impact on nutrient and carbon cycles.

Since ECM is such an important and diverse relationship, many hypotheses have been proposed about the evolution of ECM among fungi. One popular hypothesis is that ECM is a key innovation in fungi. Even if implied for individual clades of ECM fungi^11^, this hypothesis is not generally supported^12^. Instead, it seems that a few clades of fungi have experienced an increased diversification of species correlated with the transition to ECM^13,14^. The timing of origin of ECM fungal clades have also rendered some interest regarding their patterns of evolution. The oldest fossil evidence of ECM structures is only a bit more than 50 Ma old^15^, but the paucity of fossils makes this a poor estimate of the age of the symbiosis. Halling^16^ proposed that the origin of ECM lineages has been closely correlated with the origin of the plant partners, with Pinaceae in the Triassic or Jurassic, and different Angiosperm lineages, such as Fagaceae and Betulaceae, in the Late Cretaceous. Based on short branch length of many ECM clades and dating of two clades, Bruns et al.^17^ suggest that the expansion of the temperate forests (which are dominated by ECM trees, after the mid Eocene temperature maximum) has been crucial for the origin of separate ECM lineages. In support of these hypotheses individual ECM clades have been dated to the Jurassic, Late Cretaceous, and Paleogene (e.g.^11,12,18^).

The idea of the dynamics of ECM evolution has also been debated after the publication of Hibbet et al.^19^ suggesting that the evolution between ECM and non-ECM has been very dynamic, with both gains and losses. This idea has been criticized^17^, and many authors assume or support separate origins of each separate ECM lineage^11,20^. However, few have addressed the question analytically (but see^14^). There is evidence contradicting Hibbet et al.'s^19^ conclusions. Hibbett and Matheny^21^ investigated the age of fungi compared to plants and found that some nodes inferred to be ECM by Hibbett et al.^19^ are older than the oldest ECM plants. However, this conclusion is based on a few assumptions: (1) the differences in the molecular clock between plants and fungi have been modeled accurately, (2) there were no earlier ECM plants that have later gone extinct, and (3) the ECM among plants is also not dynamic with reversals from ECM to non-ECM.

The analysis of Hibbett et al.^19^ was based on a limited taxon sampling (161 species), and like most ancestral state reconstruction analyses of discrete traits, it was based on models with constant rates through time and through the tree^22^. However, the previous hypotheses on the evolution of ECM suggest that there have been changes through time^16,23^. The distribution of clades of ECM species is also not even through the fungal tree of life, which may be a reflection of higher rate of trait evolution.

Hypotheses on the importance of different time periods, differences in rates between clades, and the probability of transitions from an ECM to a non-ECM state can be represented as different models of trait evolution. Here, we compare models with different rates during different time periods with models with different rates in different clades and/or branches, and models with constant rate through the tree.

To investigate the dynamics of the evolution of ECM we compare models with zero rate from ECM to non-ECM (non-reversible) to represent the hypothesis that there have been no reversals from ECM, equal rates between ECM and non-ECM as an alternative with the same number of free parameters that clearly include the probability of reversals. We also test models where the rates between the traits are not constrained (unconstrained). To see how rate changes through the tree may affect the inference, all three models are implemented under different scenarios of changing rate through the tree. We compare models using the Akaike information criterion (AIC)^24^ and use simulations to test the fit of our data to the models, the significance of differences in AIC between models, and the probability that the model with higher AIC would get lower AIC if true^25–27^. We further perform an exploratory analysis to identify rate differences between branches in a Bayesian framework^28^. As the evolution of ECM among fungi is potentially complex and therefore needs many taxa for power of inference^1^, we use a super matrix phylogeny based on GenBank data. We show that including different rates through the tree may dramatically change our conclusion about the evolution of a trait, and caution against simplistic use of models to reconstruct trait evolution.

## Methods

### Data assembly

All sequences for the three subphyla containing ECM fungi (Agaricomycotina, Mucoromycotina, Pezizomycotina) were downloaded from GenBank. For each class, a super matrix was built from the nuc58, nucLSU, nucSSU, rpb1, and rpb2 using in-house pipeline (PifCoSm: https://github.com/RybergGroup/PifCoSm^14^, parsing gene regions with the included HMMs and linking different regions into operational taxonomic units (OTUs) based on taxon names. Each OTU was required to have nucLSU. The alignments were then iteratively cleaned by inspecting trees generated using FastTree (GTR model) (v 2.1)^29^. Taxa with long branches were identified, and the alignment was inspected and thinned to remove redundant OTUs. OTUs that based on phylogenetic placement seem miss-identified such that it would affect character coding were also removed. Only one of very similar taxa was kept, based on taxonomic annotation and branch length, while ensuring to keep ECM clades and their putative sister group^6^. The taxon sampling was then extended by including representatives of ECM clades and their putative sister taxon that were only represented by nucLSU.

Each gene was aligned separately for each class using MAFFT on the linisi option (version 7.471)^30^. The alignments for the classes were then aligned to each other using MAFFT (version 7.471)^30^. Regions with uncertain alignment were removed using trimAl (v1.2)^31^ using “gappyout” algorithm followed by manual trimming. All taxa were coded as non-ECM, ECM or uncertain (0, 1 and 01, respectively) based on literature (^6^ and others) and FUNGuild^32^.

### Phylogenetic analyses

Nucleotide substitution models were tested for each of the genes using ModelFinder in IQTree (version 2.1.1)^33^. Maximum likelihood (ML) tree was created with RAxML-NG^34^ using the respective models for each partition, twenty-five random starting trees and each subphylum constrained to be monophyletic. The tree was rooted on the branch to Mucoromycotina.

A dated phylogenetic tree was created using TreePL^35^. Cross validation was done using “leave one out cross-validation” (LOOCV) option to obtain the smoothing parameter followed by priming to find the optimization values. These values were then used as parameters to date the maximum likelihood tree created with RAxML-NG^34^ Four time points were used to calibrate the tree. These include two fossils: (1) *Paleopyrenomycites* (400 Ma; Pezizomycotina stem^36^) and (2) *Callixylon newberryi* (360 Ma; Agaricomycotina crown node^37^) (3) estimated minimum (730 Ma) and, (4) maximum (1085 Ma) times for Mucoromycotina stem node (TimeTree^38^).

### ECM evolution

An initial parsimony analysis of the ECM/non-ECM trait was done using PAUP (version 4.0a168)^39^ and phylommand-treeator (version 1.1)^40^ to explore the data. Hypotheses testing was done using a maximum likelihood approach in phylommand-treeator using a basic continuous Markov model^41^ with different constraints as outlined in the introduction. Ancestral states (non-ECM/ECM) were estimated using maximum likelihood approach in BayesTraits multistate (V3.0.2)^42^ and in phylommand-treeator with the results from BayesTraits as starting values for parameter optimizations.

### Parameter space and data simulation

The likelihood surface of parameter space was investigated using phylommand-treeator to ensure that the global maximum was reached, and to reconstruct how distinct the peak was by calculating the likelihood for multiple combinations in steps of 3e-7 from 0 to 0.006 for transition rates from non-ECM to ECM and vice versa.

The ML transitions rates and inferred state probabilities (non-ECM/ECM root) for the three models were used to generate 1000 simulated datasets. The transition rate values used for the simulation were then used as starting points to find the ML estimate of each simulated dataset.

### Rate shift in time

Models with different rates during different time periods were used to test for different rates of evolution of ECM through time. Two sets of multiple rates were tested. One was general with different rates for each geological period and the other with rate changes at five different times: (1) Mid Eocene (56 Ma), (2) start and, (3) end of Late Cretaceous (66 Ma and 100 Ma, respectively), (4) Late Triassic (237 Ma) and (5) Early Jurassic (174 Ma), based on the hypotheses by Halling^16^ and Bruns et al.^23^ The evolutionary transition rates (for non-ECM/ECM) were optimized together with rate modifiers for each time point using phylommand-treeator (Supp. data Table S1).

### Rate shift in clade

The change in rate of evolution in specific clades was also checked. Four clades were selected as candidates for deviating rates (1) Agaricomycetidae, (2) Pezizales (3) Mucoromycotina (except Endogonaceae) and (4) Thelephorales. These clades were selected as Agaricomycetidae and Pezizales contain many ECM lineages and can be expected to have a higher rate of evolution, while there are no ECM lineages outside Endogonaceae in Mucoromycotina so that part of the tree can be expected to have a lower rate of evolution^43^. Thelephorales represents the only clade with reversal from ECM to non-ECM observed in the parsimony analysis. Again, the evolutionary transition rates (for non-ECM/ECM) were optimized together with rate modifiers for each clade and multiple combinations of clades using phylommand-treeator.

All models were compared with each other using AIC. To test if the data fit the model, and if the observed difference in AIC between models were significant, one thousand datasets were simulated for each model using the ML estimates of the parameters. Simulations were performed in phylommand-treeator on the original tree, but to account for rate differences in the rate heterogeneous models the branch lengths were multiplied by the estimated rate modification parameter in phylommand-treebender. All models were then optimized on all simulated data sets using the non-modified tree. The ML score of each model on the original data was compared to the ML scores on its simulated data to estimate the probability of the observed or lower ML score given that the model is true. By comparing the observed difference in AIC score to the simulations the probability of getting this difference even if the model with higher AIC score is true was also estimated, and so was the probability of not getting a lower AIC for the model if it is true.

As Thelephorales had a reversal from ECM to non-ECM in the parsimony analysis, all models (with and without rates shifts) discussed above, except for those with separate rate in Thelephorales, were also optimized on a dataset that excluded this order.

### BayesTraits reversible jump analysis

Reverse jumps MCMC was used with variable rates command, one hundred and ten million iterations and burn-in at ten million, in BayesTraits multistate to identify the clades and branches in which the rate of evolution varies significantly. The chain was summarized with the VarRatesWebPP program (http://www.evolution.reading.ac.uk/VarRatesWebPP/) and probability of rate shifts for particular nodes and branches were summarized using var_rate_sum.pl (https://github.com/mr-y/my_bioinfPerls).

## Results

**Figure 1:**
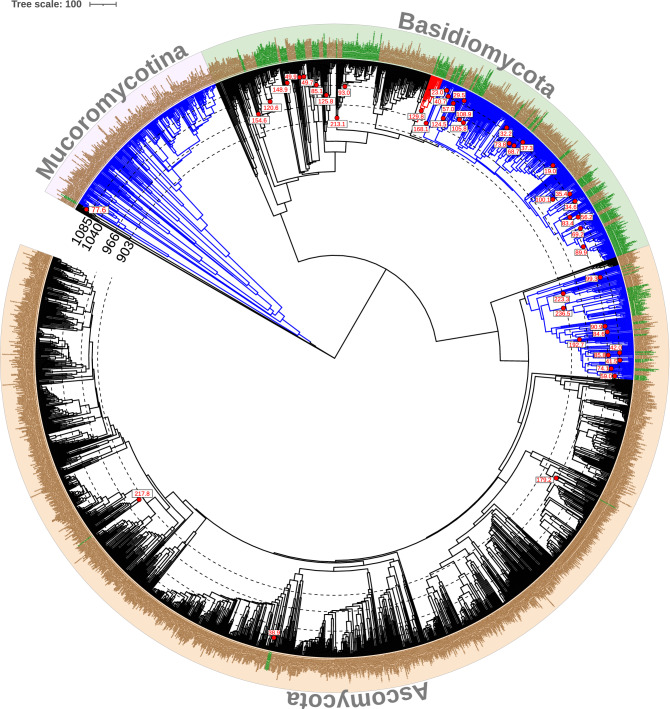
Time calibrated molecular phylogeny of ECM and non-ECM fungi. The maximum likelihood tree based on nuc58, nucLSU, nucSSU, rpb1, and rpb2 with 3225 aligned positions for 2174 OTUs. The tip labels in green are ectomycorrhizal fungi and the ones in brown are non-ectomycorrhizal. The tree was dated with TreePL using two fossils and two estimated minimum and maximum times: (1) *Paleopyrenomycites* (400 Ma; Pezizomycotina stem) and (2) *Callixylon newberryi* (360 Ma; Agaricomycotina stem node) (3) estimated minimum (730 Ma) and, (4) maximum (1085 Ma) times for Mucoromycotina stem node. The dotted lines represent the distance from the root in terms of million years for four of the shifting points for rate shift in time with five geological time points (903, 966, 1040, 1074 and 1085 MYA) analyses. The clade in red is Thelephorales. The ones in blue are Agaricomycetidae, Pezizales, and Mucoromycotina (except Endogonacae). The numbers on the outside of the ECM taxon labels represent the divergence time for the crown ECM nodes. The scale bar shows the geological time scale in million years. The figure was generated using iTOL (v6) (https://itol.embl.de/).

Removal of uncertain sites from the concatenated alignment of five genes resulted in 3225 aligned nucleotide positions for 2174 OTUs, which were used for the maximum likelihood tree and the dated phylogeny. All OTUs had nucLSU, whereas 1706 had nuc58, 1654 had rpb2, 1345 had rpb1 and 1038 had nucSSU genes. The topology was largely in agreement with previous estimates. The root of the tree was dated to 1140 Ma and the split between Agaricomycetes and Pezizomycetes to be 747 Ma ago (Fig. 1, Supp. data Table S2).

### Model comparisons using ML

**Figure 2:**
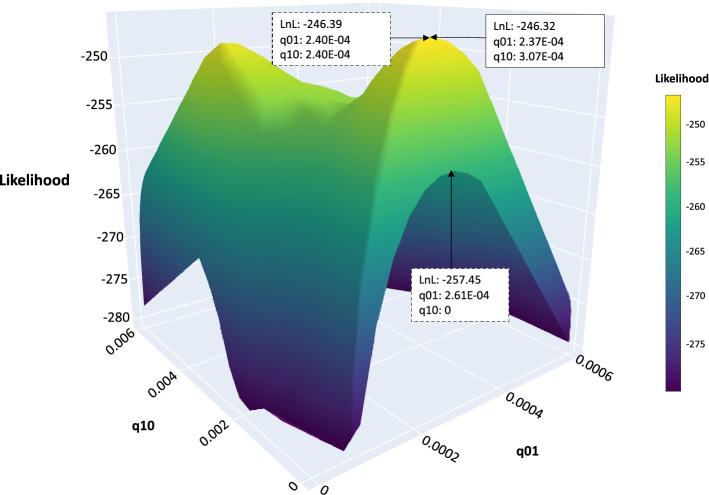
Likelihood surface for rate homogeneous models. The axes show rate of change from non-ectomycorrhizal to ectomycorrhizal (q01) and vice versa (q10) and the likelihood value. The local peaks are shown in green and the global maximum is shown in yellow. The boxes show the likelihood at the global maxima with optimal transition rates (q01 and q10) for unconstrained (solid box), equal and non-reversible models (dotted boxes).

Comparison of ML scores on the real data with the score on the simulated data did not reject that the pattern of ECM/non-ECM taxa in the phylogeny could be generated by any of the models (*P* > 0.16). Among the rate homogenous models, the equal rates model gets the lowest AIC (Supp. data Table S3), but the unconstrained model could not be rejected (*P* = 0.22; Supp. Data. Table S4), and it estimates that the rate of evolution from ECM to non-ECM is higher than the reverse (Supp. data Table S3). Looking at the likelihood landscape, the likelihood clearly drops as the rate from non-ECM to ECM deviate drastically from the ML estimate. However, on the axis for the rate from ECM to non-ECM, a ridge is formed reaching an order of magnitude higher rate than the ML estimate, but it drops steeply towards rate zero (Fig. 2) and both the unconstrained and equal rate models are significantly better than the non-reversible model (*P* = 0 in both cases). The optimal value was found for each model when enough searches were done in BayesTraits Multistate or appropriate starting values were provided in phylommand-treeator. However, there were local peaks that individual runs got stuck in.

Implementation of models with rate shifts in clades show that unless Thelephorales is one of the clades with a separate rate, the equal rate or unconstrained model gets the lowest AIC, but neither is significantly lower than the other. However, the AIC for the non-reversible is significantly higher. The non-reversible model gets the lowest AIC when implementing a rate shift in Thelephorales, and it is significantly lower than the equal rate model but not the unconstrained model (Supp. data Tables S3 and S4).

For the models with rate change in time, the unconstrained model was significantly better than the equal rate model, which in turn was significantly better than the non-reversible model. Each of the three had lower AIC values when implementing separate rate for five time periods than for each time period, but not significantly so. The simulations also show that there is a high probability that the five time period model gets lower AIC, even if the model with different rates in each time period is true, for all of the unconstrained (*P* = 0.99), equal rate (*P* = 1.0), and non-reversals models (*P* = 1.0). The unconstrained model suggests a higher rate of evolution to non-ECM from ECM than the reverse and also an ECM root with both different rates under five periods and under each period. The unconstrained model with different rates under five periods shows the highest rate of evolution during start and end of Late Cretaceous (27.64 and 20.603) and a low rate of evolution close to the tips (2.91) and the model with different rates during each period also shows the highest rate of evolution during Late Cretaceous (4550.2) and the lowest during Late Jurassic (0.077), and varies through the other time periods with no clear pattern (Supp. data Table S1).

**Figure 3:**
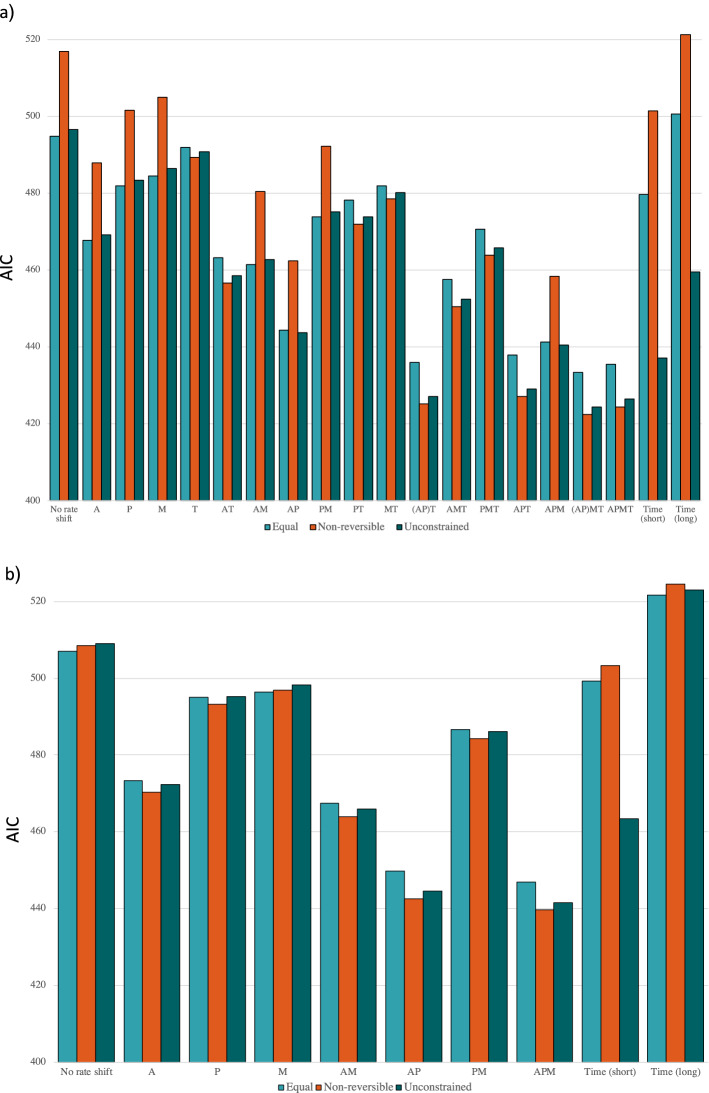
Comparison of AIC scores amongst different models for (**a**) dataset with Thelephorales and (**b**) dataset without Thelephorales. The y-axis show the AIC score for each model shown on the x-axis where models with rate shifts are specified with either symbol or combinations of symbols where A = Agaricomycetidae, P = Pezizales, M = Mucoromycotina (except Endogonacae), T = Thelephorales, Time (short) = time with 5 geological time points, T(long) = time with all geological time points. Symbols in braces mean that that the taxa were constrained to have the same rates.

Overall, the non-reversible model with rate shift for all four clades with the rate constrained to be equal for Agaricomycetidae and Pezizales has the lowest AIC score. All the ten models with lowest AIC score included a rate shift in Agaricomycetidae, Pezizales and Thelephorales. However, there were several models without a rate shift in Thelephorales for which the unconstrained model did not have significantly lower AIC (Fig. 3a, Supp. data Tables S3 and S4).

### Model comparisons using ML without Thelephorales

For the dataset without Thelephorales, the equal rate model still has the lowest AIC score amongst the three models when assuming a constant rate through the tree, and also when having separate rate for only Mucoromycotina (except Endogonaceae). For the other models with rate shifts in clades the non-reversible model gets lowest AIC. For the model with different rates in each period, the equal rate model gets lowest AIC. For the models with rate shift during five time periods, the unconstrained rate model gets the lowest AIC with a rate estimate of going from ECM to non-ECM as higher than the opposite direction. Unlike for the data with Thelephorales the ancestor at the root is reconstructed as most likely being non-ECM for unconstrained model for each period. The non-reversible model with rate shift in clade for the three clades (Agaricomycetidae, Pezizales and Mucoromycotina (except Endogonaceae)) has the lowest AIC score of all tested models (Fig. 3b, Supp. data Table S5).

### Bayesian analysis

The BayesTraits analysis suggests models with many more parameters to account for rate differences throughout the tree. The median number of rate changes at nodes was 90 (95% credibility interval 73–109), and the median number of branches with separate rate was 184 (95% credibility interval 158–210). The median rates for each branch reflect a higher rate of evolution in Pezizales, and a much higher rate for most of Thelephorales (but not necessarily uniform rate through the respective clade). It not only suggests a higher rate for Agaricomycetidae but also for all of Agaricomycetes, with several branches with even higher rate, while some do not have such a high rate. The rest of the tree mostly has a median rate close to 1, but some branches have higher median rate estimates, though not as high as for most of Agaricomycetes and Pezizales (Supp. data Figure S1). Nevertheless, it is difficult to pinpoint specific nodes with rate changes. The nodes with highest posterior probability (PP) of a rate change delimits Thelephorales except Amaurodon (a non-ECM sister to the rest of the Thelephorales; PP = 0.73), Pezizales (PP = 0.59), and Agaricomycetes except Auriculariales (a non-ECM sister to the rest of the Agaricomycetes in our phylogeny; PP = 0.46). No branch had a PP above 0.08 for a branch specific rate. There is no time period with consistently higher rates through the tree, but the standard deviation of all branch rate estimates is high. The estimated posterior probability (PP) for the rate from ECM to non-ECM to be higher than the reverse is 0.00, for the former to be less than 10% as high as the latter is 0.90, and that the rate from ECM to non-ECM is zero is 0.47.

## Discussion

The result that the rate of evolution to non-ECM from ECM is at least about as high as the rate to ECM from non-ECM, if assuming the same rate throughout the tree (Supp. data Table S3), is contrary to what is generally postulated in the field (Bruns and Shefferson 2004; Tedersoo et al. 2012). However, comparison with models that allow different rates in different clades (Supp. data Tables S1, S3 and S4) show that the non-reversible model is the best fitting model in many scenarios, including the model with the lowest AIC score. However, the model with lowest AIC is not significantly better than all the other models and while many of the alternative models inferred a higher rate of evolution from non-ECM to ECM some inferred the opposite.

A key to the difference in inference between different models is how the rate in Thelephorales is treated. Parsimony analysis indicated that there has been a reversal from ECM to non-ECM in Thelephorales, but this may instead be explained by no reversals to non-ECM and a very high rate of evolution to ECM in this clade (Supp. data Table S3), which then gives a model that fits better with the patterns in the rest of the tree.

Thelephorales is often a major component of ECM fungal communities^44^ but represent an under-sampled part of the fungal tree of life and most taxa in the clade were only represented by nucLSU. The position of the branch with the reversal to non-ECM is consequently not well supported in our analysis, and there is no comprehensive reference phylogeny to compare our phylogeny with. Tedersoo et al.^20^ show different phylogenetic relationships than those reported here, but only refer to unpublished data, while Sanchez-Garcia et al.^14^ also find a transition from ECM in this clade. As the phylogeny is uncertain, it may be that the parameter estimates are skewed by a miss-representation of the evolutionary relationships in this order. However, excluding Thelephorales from the analyses still gives the lowest AIC to the equal rates model if the rate is assumed to be constant through the tree (Fig. 3b, Supp. data Table S5) and the results still point at the importance of accounting for possible differences in rates through the tree.

There are some lineages that have been suggested to be nested within ECM lineages and for which the nutritional mode is not very clear. They can form ECM or ECM like structures (for example^45,46^), but may be parasitic rather than mutualistic or they also form parasitic relations^45,47^. These lineages were not included here. To keep the models simple, the non-ECM category was not further resolved between saprotrophic and biotrophic nutritional modes. However, previous studies have suggested that most ECM lineages have saprotrophic ancestors^48^.

Previous hypotheses on the evolution of ECM have focused mainly on what time periods that have been most important^16,23^, but our results indicate that rate shifts in clades are more important as all models that include different rate than the base rate for Agaricomycetidae and Pezizales have lower AIC scores than any model with rate shift in time. This conclusion is further strengthened when excluding Thelephorales from the analysis. This finding suggests that changes in individual fungal lineages, rather than wide reaching external events, have mattered the most for the evolution of the present-day ECM fungal diversity. Such changes may have happened before the evolution of ECM symbiosis (age of Pezizales 368 Ma, and Agaricomycetidae 230 Ma), but what the changes constitute remains unknown.

Even if changes in individual fungal lineages are most important for the observed pattern in the phylogeny, it is possible that external events created the opportunities for the fungal lineages with the right traits to take advantage of them. This is at least evident from the need of an ECM plant partner. While the unconstrained model with rate shifts in every time period could not be confidently rejected (*P* = 0.78), the rate parameter estimates for this model do not lend clear support to neither of the two hypotheses of specific time periods with higher rate of evolution. It furthermore suggests the root to be ECM which would make the oldest ECM fungi not only older than the oldest known ECM plant lineage, but also older than the first plants on land, which seems unlikely.

There are, however, some weaknesses to the method used. It may be more difficult to get reliable parameter estimates for older time periods since there are fewer separate lineages in them that are represented in the tree of present-day taxa. Furthermore, the specific rate estimates for the time periods depend on our dating of the nodes. Our dating analysis gives older splits for between the classes than many other studies (e.g.^49,50^) but younger than some (e.g.^51,52^). For individual ECM clades, the estimates may be either younger or older compared to other sources, depending on clade and reference (e.g.^11,12,53,54^). This reflects that the dating of clade ages in fungi are still very uncertain at large phylogenetic scales^55^ and even more so for specific clades.

Models that include both rate shifts in clades and time may explain the evolution of ECM even better than the models tested here. Too complex models may however easily overfit the data and may not be possible to distinguish from simpler models. This is exemplified here by the fact that the model with different rates in all geological time periods could not be separated from the model with rate shifts only in periods that have been suggested to be of importance, based on our simulations. The likelihood landscapes may also become complex, which makes it difficult to find the maximum likelihood estimates of the parameters. This is a risk even for the rate homogenous models, and a potential limitation for the most complex models that we implemented. A Bayesian framework may work better for such models^56^, and could also include uncertainty in the phylogenetic estimates. We ran the rate variable reversible jump option in BayesTraits and found support for rate heterogeneity and a lower rate of evolution from ECM to non-ECM than the reverse. However, as the suggested models are complex, and there is no strong support for any particular model and parameter estimates, it is difficult to summarize the analyses into a specific supported hypothesis to compare to others. In any case, it does not give any clear support for models with shifts both in time and clades, but for shifts in clades. As no specific model is supported, and the flat prior on nodes for rate shifts may bias towards models with many parameters, just because there are many more such models (e.g. there is only one model with homogenous rates through the tree), the analyses should not be taken as support for models with hundreds of parameters. The averaging rates over models largely agree with our ML analysis but suggest that there may be models that we did not test that would be even better fit to the data. As generating a reasonable estimate of the posterior probability distribution for a dated phylogeny of the size used in this paper is too slow to be feasible, we relied on the phylogeny produced in a maximum likelihood setting, and not a full Bayesian approach.

## Conclusions

Our analyses suggest that reversals from ECM to non-ECM are probably rare and that changes in individual lineages have been more important than individual time periods for the evolution of ECM fungal diversity. Nevertheless, the ML analysis does not entirely exclude the likelihood of ECM to non-ECM transitions and we acknowledge that other more complex models may give other results. However, despite accumulation of more and more data for even more inclusive taxon sampling, we doubt that only analyses of the patterns of ECM fungi in the phylogeny will be able to give a conclusive answer. All models are simplifications of reality but over simplistic analyses may give results that are likely to be incorrect. It is therefore important to carefully evaluate the assumptions of models and their alternatives, such as rate changes through the phylogeny, as this may fundamentally change the inference of the evolutionary process^57^. It further seems like, even in the presence of large-scale phylogenies, the inference of trait evolution may be greatly affected by the resolution of a few nodes.

## Supplementary Information

Supplementary Information 1.

Supplementary Information 2.

Supplementary Information 3.

Supplementary Information 4.

Supplementary Information 5.

Supplementary Information 6.

Supplementary Information 7.

## Data Availability

The data underlying this article are available in the Dryad Digital Repository at https://doi.org/10.5061/dryad.wdbrv15qd..
