# Supplementary figures and images for: Impact of model assumptions on the inference of the evolution of ectomycorrhizal symbiosis in fungi
Source: Sci Rep. 2022 Dec 21;12:22043. doi: 10.1038/s41598-022-26514-2 (PMC9772227; doi:10.1038/s41598-022-26514-2)

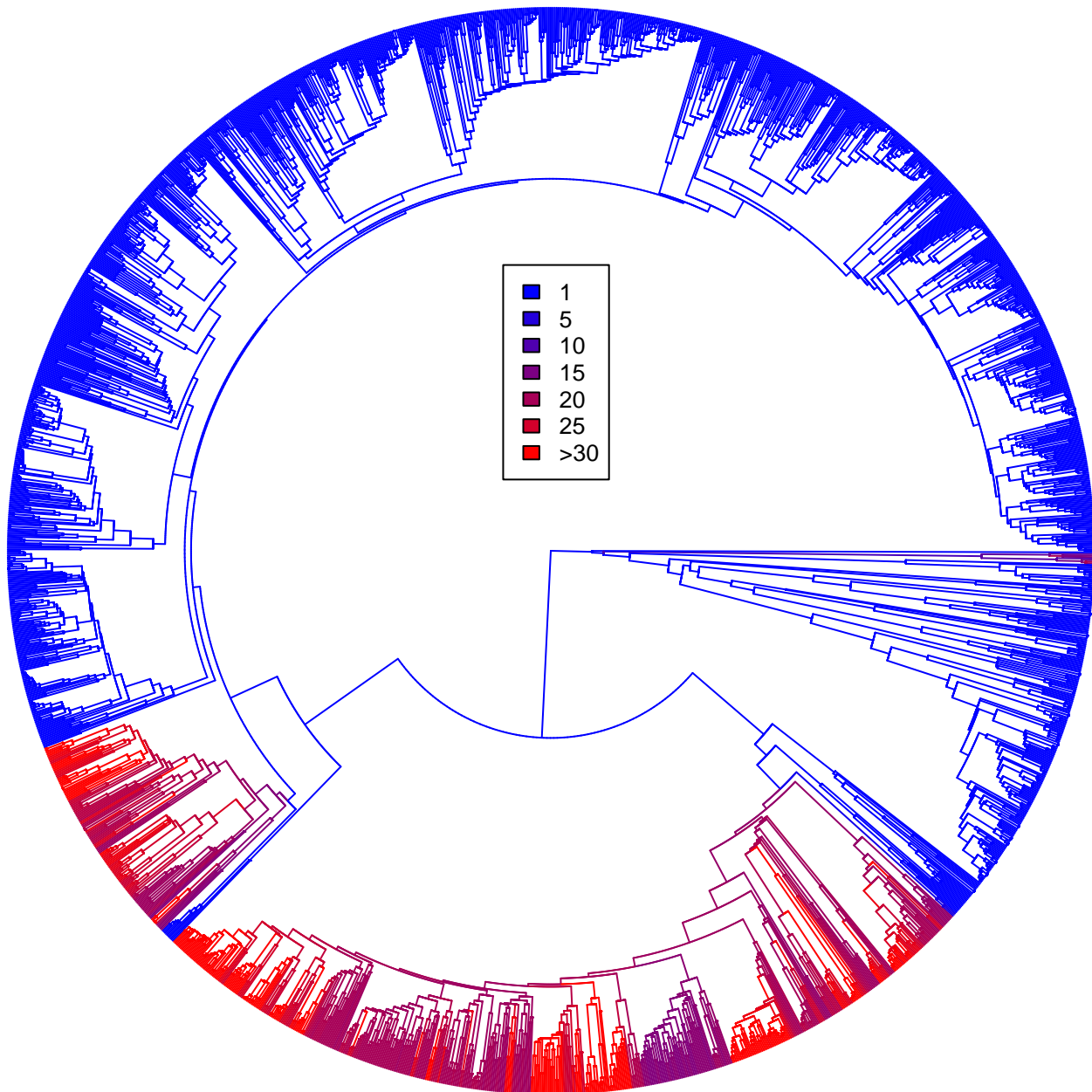

Supplement: Supplementary file 1 — Supplementary Information 1. [file 41598_2022_26514_MOESM1_ESM.pdf]
