# Supplementary material for: Impact of model assumptions on the inference of the evolution of ectomycorrhizal symbiosis in fungi
Source: Sci Rep. 2022 Dec 21;12:22043. doi: 10.1038/s41598-022-26514-2 (PMC9772227; doi:10.1038/s41598-022-26514-2)
Supplement: Supplementary file 2 — Supplementary Information 2. [file 41598_2022_26514_MOESM2_ESM.docx]

**Supplementary data:**

**Figure S1: Tree with branches colored by median rate based on the estimated posterior distribution thinned down to 10 000 trees. The colors on the branches represent rates, with blue** representing slow rates and red fast rates. For resolution, in the range where most branches are, the scale tops out at 30, even if the median rate may reach 637. The topology is the same as in Figure 1, and taxonomic groups can be found in that figure.

**Table S1: Rates of evolution for models with rate shift in time for (a) five time points and (b) each geological time period.** The tables show the name of the time period in the first column followed by the start time in million years from the present in the second column. The third column shows the “rate modifier” which shows the rate of evolution and is the value that each branch is multiplied by until the time from the root of the tree (as given to phylommand-treeator) specified in the last column. A higher “rate modifier” value depicts a higher rate of evolution at a certain time period.

**Table S2: Crown dates for ECM lineages.** Molecular dating was done using TreePL using two fossils and two estimated minimum and maximum times: (1) *Paleopyrenomycites* (400 Mya; Pezizomycotina stem) and (2) *Callixylon newberryi* (360 Mya; Agaricomycotina crown node) (3) estimated minimum (730 Mya) and, (4) maximum (1085 Mya) times for Mucoromycotina stem node. In case the OTUs are not monophyletic, all crown dates are given separated by a slash.

**Table S3: Simulation based comparison of AIC amongst all models.** The table show the comparison of AIC between all models based on simulations. For each comparison the simulations are based on the model given in the column. The columns are arranged from lower to higher AIC values, based on the observed data. The cell where the model is compared with itself (grey cell) show the probability of the observed or lower ML value based on data simulated using the same model. When the model on the row is compared to a model with lower AIC (left of grey cell) it gives the probability of observing a lower AIC for the model in the column, i.e. the probability of considering the true model as better. When the model on the row is compared to a model with higher AIC (right of the grey cell) the probability of the observed, or larger, ΔAIC between the models is given, i.e. the probability of the observed difference even if the model with higher AIC is true. E=equal, N=non-reversible, U=unconstrained. Models with rate shifts are specified with either symbol or combinations of symbols where A=Agaricomycetidae, P=Pezizales, M=Mucoromycotina (except Endogonacae), T=Thelephorales, Time (5)=time with 5 geological time points, T(21)= time with all geological time points. Symbols in braces mean that that the taxa were constrained to have the same rates.

**Table S4: Ancestral state reconstruction using equal, non-reversal and unconstrained models with and without rate shifts.** The “likelihood” column gives the likelihood value for a certain model (specified at the top of each section in dark grey) given the rate of change from non-ectomycorrhizal to ectomycorrhizal and vice versa (“Rate”). The “AIC” column gives the AIC score for each model based on the likelihood and number of parameters written in “#Parameter” column. Root (ECM/non-ECM) is the probability of having an ectomycorrhizal respectively non-ectomycorrhizal root. For models with rate shift in clade, the column with “Clades” specifies the clades being tested and the column with “Rate modifier” gives the value for the rate of evolution of each clade under the specified model. For models with rate shift in time, the column with “Time” shows the time points used for the analyses. E=equal, N=non-reversible, U=unconstrained models. The lowest AIC value for each of the tree models under different models of rate shifts is marked in green, and the overall lowest AIC in darker green. The top row gives the “Rate” values obtained using BayesTraits that were used as starting points for the analyses with no rate shift with phylommand-treeator. All the other analyses were conducted using phylommand-treeator.

**Table S5: Ancestral state reconstruction using equal, non-reversal and unconstrained models with and without rate shifts for dataset without Thelephorales.** The “likelihood” column gives the likelihood value for a certain model (specified at the top of each section in dark grey) given the rate of change from non-ectomycorrhizal to ectomycorrhizal and vice versa (“Rate”). The “AIC” column gives the AIC score for each model based on the number of parameters written in “#Parameter” column. Root (ECM/non-ECM) is the probability of having an ectomycorrhizal or non-ectomycorrhizal root. For models with rate shift in clade, the column with “Clades” specifies the clades being tested and the column with “Rate modifier” gives the value for the rate of evolution of each clade under the specified model. For models with rate shift in time, the column with “Time” shows the time points used for the analyses. E=equal, N=non-reversible, U=unconstrained models. The lowest AIC value for each of the tree models under different models of rate shifts is marked in green, and the overall lowest AIC in darker green. The top row gives the “Rate” values obtained using BayesTraits that were used as starting points for the analyses with no rate shift with phylommand-treeator. All the other analyses were conducted using phylommand-treeator.
