# Supplementary material for: Impact of model assumptions on the inference of the evolution of ectomycorrhizal symbiosis in fungi
Source: Sci Rep. 2022 Dec 21;12:22043. doi: 10.1038/s41598-022-26514-2 (PMC9772227; doi:10.1038/s41598-022-26514-2)
Supplement: Supplementary file 3 — Supplementary Information 3. [file 41598_2022_26514_MOESM3_ESM.pdf]

Table S1: Rates of evolution for models with rate shift in time for (a) five time points and (b) each geological time period. The tables show the name of the time period in the first column followed by the start time in million years from the present in the second column. The third column shows the "rate modifier" which shows the rate of evolution and is the value that each branch is multiplied by until the time from the root of the tree (as given to phylommand-treeator) specified in the last column. A higher "rate modifier" value depicts a higher rate of evolution at a certain time period.

(a)		Period	Time (Mya)	Rate modifier	Distance from root (Mya)
Five time points	Equal	Late Triassic (start)	237	2.77E+00	903.209
		Early Jurassic (end)	174.1	1.11E+01	966.109
		Late Cretaceous (start)	100	1.21E+01	1040.21
		Late Cretaceous (end)	66	1.35E+01	1074.21
		Mid Eocene	55.6	9.20E+00	1084.61
	Non-reversible	Late Triassic (start)	237	2.29E+00	903.209
		Early Jurassic (end)	174.1	8.57E+00	966.109
		Late Cretaceous (start)	100	1.05E+01	1040.21
		Late Cretaceous (end)	66	1.50E+01	1074.21
		Mid Eocene	55.6	8.27E+00	1084.61
	Unconstrained	Late Triassic (start)	237	2.72E+00	903.209
		Early Jurassic (end)	174.1	9.56E+00	966.109
		Late Cretaceous (start)	100	2.76E+01	1040.21
		Late Cretaceous (end)	66	2.06E+01	1074.21
		Mid Eocene	55.6	2.91E+00	1084.61

(b)		Period	Time (Mya)	Rate modifier	Distance from root (Mya)
Each geological time point	Equal	Neoproterozoic	1000	2.97E-02	140.21
		Cambrian	541	4.74E-03	599.209
		Ordovician	485.4	2.12E-02	654.81
		Silurian	443.8	1.30E-02	696.409
		Devonian	419.2	8.50E-03	721.01
		Carboniferous	358.9	8.33E-03	781.31
		Permian	298.9	6.71E+01	841.31
		Triassic (lower)	251.9	6.04E-04	888.307
		Triassic (middle)	247	1.25E+03	893.01
		Triassic (upper)	237	4.11E-01	903.209
		Jurassic (lower)	201.3	1.08E-03	938.909
		Jurassic (middle)	174.1	3.10E+02	966.109
		Jurassic (upper)	163.5	8.69E-03	976.709
		Cretaceous (lower)	145	1.16E-03	995.209
		Cretaceous (upper)	100.5	1.73E+02	1039.71
		Paleocene	66	1.59E+02	1074.21
		Eocene	56	5.64E-05	1084.21
		Oligocene	33.9	7.34E+01	1106.31
		Miocene	23.03	8.50E-03	1117.18
		Pliocene	5.33	5.14E-04	1134.88
		Pleistocene	2.58	1.45E-04	1137.63
	Non-reversible	Neoproterozoic	1000	1.40E+02	140.21
		Cambrian	541	5.99E+02	599.209
		Ordovician	485.4	6.55E+02	654.81
		Silurian	443.8	6.96E+02	696.409
		Devonian	419.2	7.21E+02	721.01
		Carboniferous	358.9	7.81E+02	781.31
		Permian	298.9	8.41E+02	841.31
		Triassic (lower)	251.9	8.88E+02	888.307
		Triassic (middle)	247	8.93E+02	893.01
		Triassic (upper)	237	9.03E+02	903.209
		Jurassic (lower)	201.3	9.39E+02	938.909
		Jurassic (middle)	174.1	9.66E+02	966.109
		Jurassic (upper)	163.5	9.77E+02	976.709
		Cretaceous (lower)	145	9.95E+02	995.209
		Cretaceous (upper)	100.5	1.04E+03	1039.71
		Paleocene	66	1.07E+03	1074.21
		Eocene	56	1.08E+03	1084.21
		Oligocene	33.9	1.11E+03	1106.31
		Miocene	23.03	1.12E+03	1117.18
		Pliocene	5.33	1.13E+03	1134.88
		Pleistocene	2.58	1.14E+03	1137.63
	Unconstrained	Neoproterozoic	1000	1.22E+03	140.21
		Cambrian	541	2.40E+00	599.209
		Ordovician	485.4	6.13E+02	654.81
		Silurian	443.8	5.21E+01	696.409
		Devonian	419.2	1.30E+01	721.01
		Carboniferous	358.9	1.60E+03	781.31
		Permian	298.9	6.23E+01	841.31
		Triassic (lower)	251.9	2.50E+00	888.307
		Triassic (middle)	247	1.05E+01	893.01
		Triassic (upper)	237	1.93E+02	903.209
		Jurassic (lower)	201.3	1.92E+03	938.909
		Jurassic (middle)	174.1	8.72E+02	966.109
		Jurassic (upper)	163.5	7.68E-02	976.709
		Cretaceous (lower)	145	4.32E+03	995.209
		Cretaceous (upper)	100.5	4.55E+03	1039.71
		Paleocene	66	2.49E+03	1074.21
		Eocene	56	3.34E+01	1084.21
		Oligocene	33.9	8.06E+02	1106.31
		Miocene	23.03	1.20E+00	1117.18
		Pliocene	5.33	2.22E+00	1134.88
		Pleistocene	2.58	2.56E+00	1137.63
