# Supplementary material for: Impact of model assumptions on the inference of the evolution of ectomycorrhizal symbiosis in fungi
Source: Sci Rep. 2022 Dec 21;12:22043. doi: 10.1038/s41598-022-26514-2 (PMC9772227; doi:10.1038/s41598-022-26514-2)
Supplement: Supplementary file 4 — Supplementary Information 4. [file 41598_2022_26514_MOESM4_ESM.pdf]

Table S2: Crown dates for ECM lineages. Molecular dating was done using TreePL using two fossils and two estimated minimum and maximum times: (1) *Paleopyrenomycites* (400 Mya; Pezizomycotina stem) and (2) *Callixylon newberryi* (360 Mya; Agaricomycotina crown node) (3) estimated minimum (730 Mya) and, (4) maximum (1085 Mya) times for Mucoromycotina stem node. In case the OTUs are not monophyletic, all crown dates are given separated by a slash.

Lineages of ECM fungi	Age (Mya)
**BASIDIOMYCOTA**	
**AGARICALES**	
/amanita	69.27
/catathelasma	73.8
/cortinarius	69.2
/descolea	35.4
/entoloma	19.03
/hebeloma-alnicola	56.2
/hydropus	124.2/108.1
/hygrophorus	32.2
/inocybe	89.9
/laccaria	34.6
/paralyophyllum	37.3
/tricholoma	68.7
**ATHELIALES**	
/amphinema-tylospora	40.7/168.1
/byssocorticium	17.96
/piloderma	23.04
**BOLETALES**	
/austropaxillus	57.04
/boletus	117.3
/paxillus-gyrodon	108.9/76.95
/pisolithus-scleroderma	59.5
/suillus-rhizopogon	124.5
**CANTHARELLALES *S. LATO***	
/cantharellus	49.7
/clavulina	85.1
/ceratobasidium	421.2
**GOMPHALES**	
/clavariadelphus	154.6
/gautieria	38.5
**HYMENOCHAETALES**	
/coltricia	125.8
**HYSTERANGIALES**	
/hysterangium	120.6
**RUSSULALES**	
/albatrellus	213.1
/russula-lactarius	93.03
**SEBACINALES**	
/sebacina	63.6
**THELEPHORALES**	
/boletopsis	103.9
/hydnellum-sarcodon	76.8
/pseudotomentella	76.8
/tomentella-thelephora	95.1
/tomentellopsis	94.8
**ASCOMYCOTA**	
**DOTHIDEOMYCETES INCERTAE SEDIS**	
/cenococcum	38.95
**EUROTIALES**	
/elaphomyces	170.7
**PEZIZALES**	
/genea-humaria	74.1
/geopora	41.5
/hydnotrya	223.3
/otidea	59
/pulvinula	84.6
/sowerbyella	42
/sphaerosporella	85.8
/tarzetta	48.8
/terfezia-peziza depressa	70.4
/tuber-helvella	162.9/123.2
/wilcoxina	192.7
**SORDARIALES**	
**/sordariales1**	217.8
**ZYGOMYCOTA**	
**ENDOGONALES**	
/endogone1 (*Endogone pisiformis*)	66.2
/endogone2 (*Endogone flammicorona*)	17.4
