# Supplementary material for: Impact of model assumptions on the inference of the evolution of ectomycorrhizal symbiosis in fungi
Source: Sci Rep. 2022 Dec 21;12:22043. doi: 10.1038/s41598-022-26514-2 (PMC9772227; doi:10.1038/s41598-022-26514-2)
Supplement: Supplementary file 7 — Supplementary Information 7. [file 41598_2022_26514_MOESM7_ESM.pdf]

	No rate shift						
	# Parameters	Likelihood	AIC	Rate (non-ECM to ECM)	Rate (ECM to non-ECM)	Root (non-ECM)	Root (ECM)
**E**	**1**	-252.53	507.05	2.46E-04	2.46E-04	9.98E-01	2.40E-03
**N**	**1**	-253.24	508.47	2.55E-04	0.00E+00	1.00E+00	0.00E+00
**U**	**2**	-252.52	509.04	2.47E-04	2.22E-04	9.98E-01	1.92E-03

	Rate shift (clade: Agricomycetidae)							
	# Parameters	Likelihood	AIC	Rate modifier	Rate (non-ECM to ECM)	Rate (ECM to non-ECM)	Root (non-ECM)	Root (ECM)
**E**	**2**	-234.68	473.35	6.70E+00	1.51E-04	1.51E-04	9.99E-01	8.37E-04
**N**	**2**	-233.18	470.36	7.36E+00	1.56E-04	0.00E+00	1.00E+00	0.00E+00
**U**	**3**	-233.18	472.36	7.36E+00	1.56E-04	6.54E-13	1.00E+00	1.51E-20

	Rate shift (clade: Pezizales)							
	# Parameters	Likelihood	AIC	Rate modifier	Rate (non-ECM to ECM)	Rate (ECM to non-ECM)	Root (non-ECM)	Root (ECM)
**E**	**2**	-245.56	495.11	4.33E+00	2.00E-04	2.00E-04	9.98E-01	1.52E-03
**N**	**2**	-244.59	493.18	4.97E+00	2.05E-04	0.00E+00	1.00E+00	0.00E+00
**U**	**3**	-244.59	495.18	4.97E+00	2.05E-04	0.00E+00	1.00E+00	0.00E+00

	Rate shift (clade: Mucormycotina (except Endogonacae))							
	# Parameters	Likelihood	AIC	Rate modifier	Rate (non-ECM to ECM)	Rate (ECM to non-ECM)	Root (non-ECM)	Root (ECM)
**E**	**2**	-246.20	496.39	0.00E+00	2.80E-04	2.80E-04	9.97E-01	3.14E-03
**N**	**2**	-246.44	496.88	0.00E+00	2.93E-04	0.00E+00	1.00E+00	0.00E+00
**U**	**3**	-246.09	498.18	0.00E+00	2.84E-04	1.88E-04	9.99E-01	1.37E-03

Table 5: Ancestral state reconstruction using equal, non-reversal and unconstrained models with and without rate shifts for dataset without Thelephorales. The "likelihood" column gives the likelihood value for a certain model (specified at the top of each section in dark grey) given the rate of change from non-ectomycorrhizal to ectomycorrhizal and vice versa ("Rate"). The "AIC" column gives the AIC score for each model based on the number of parameters written in "#Parameter" column. Root (ECM/non-ECM) is the probability of having an ectomycorrhizal or non-ectomycorrhizal root. For models with rate shift in clade, the column with "Clades" specifies the clades being tested and the column with "Rate modifier" gives the value for the rate of evolution of each clade under the specified model. For models with rate shift in time, the column with "Time" shows the time points used for the analyses. E=equal, N=non-reversible, U=unconstrained models. The lowest AIC value for each of the tree models under different models of rate shifts is marked in green, and the overall lowest AIC in darker green. The top row gives the "Rate" values obtained using BayesTraits that were used as starting points for the analyses with no rate shift with phylommand-treeator. All the other analyses were conducted using phylommand-treeator.

	Rate shift (Agricomycetidae, Mucormycotina (except Endogonacae))								
	# Parameters	Likelihood	AIC	Clade	Rate modifier	Rate (non-ECM to ECM)	Rate (ECM to non-ECM)	Root (non-ECM)	Root (ECM)
**E**	**3**	-230.72	467.43	Agricomycetidae	5.79E+00	1.74E-04	1.74E-04	9.99E-01	1.14E-03
				Mucoromycotina	6.02E-08				
**N**	**3**	-229.00	463.99	Agricomycetidae	6.30E+00	1.82E-04	0.00E+00	1.00E+00	0.00E+00
				Mucoromycotina	0.00E+00				
**U**	**4**	-229.00	465.99	Agricomycetidae	6.30E+00	1.82E-04	0.00E+00	1.00E+00	0.00E+00
				Mucoromycotina	0.00E+00				

	Rate shift (Agaricomycetidae, Pezizales)								
	# Parameters	Likelihood	AIC	Clade	Rate modifier	Rate (non-ECM to ECM)	Rate (ECM to non-ECM)	Root (non-ECM)	Root (ECM)
**E**	**3**	-221.84	449.69	Agricomycetidae	1.11E+01	9.07E-05	9.07E-05	1.00E+00	2.92E-04
				Pezizales	9.53E+00				
**N**	**3**	-218.30	442.60	Agricomycetidae	1.24E+01	9.27E-05	0.00E+00	1.00E+00	0.00E+00
				Pezizales	1.10E+01				
**U**	**4**	-218.30	444.60	Agricomycetidae	9.27E-05	9.27E-05	8.19E-14	1.00E+00	2.33E-22
				Pezizales	1.24E+01				
					1.10E+01				

	Rate shift (Pezizales, Mucormycotina (except Endogonacae)								
	# Parameters	Likelihood	AIC	Clade	Rate modifier	Rate (non-ECM to ECM)	Rate (ECM to non-ECM)	Root (non-ECM)	Root (ECM)
**E**	**3**	-240.35	486.71	Pezizales	3.76E+00	2.30E-04	2.30E-04	9.98E-01	2.05E-03
				Mucoromycotina	0.00E+00				
**N**	**3**	-239.10	484.20	Pezizales	4.29E+00	2.38E-04	0.00E+00	1.00E+00	0.00E+00
				Mucoromycotina	0.00E+00				
**U**	**4**	-239.10	486.20	Pezizales	4.29E+00	2.38E-04	9.62E-14	1.00E+00	3.32E-22
				Mucoromycotina	0.00E+00				

	Rate shift (Agricomycetidae, Pezizales, Mucormycotina (except Endogonacae))								
	# Parameters	Likelihood	AIC	Clade	Rate modifier	Rate (non-ECM to ECM)	Rate (ECM to non-ECM)	Root (non-ECM)	Root (ECM)
**E**	**4**	-219.42	446.85	Agricomycetidae	9.47E+00	1.07E-04	1.07E-04	1.00E+00	4.07E-04
				Pezizales	8.11E+00				
				Mucoromycotina	0.00E+00				
**N**	**4**	-215.79	439.59	Agricomycetidae	1.05E+01	1.10E-04	0.00E+00	1.00E+00	0.00E+00
				Pezizales	9.30E+00				
				Mucoromycotina	0.00E+00				
**U**	**5**	-215.79	441.59	Agricomycetidae	1.05E+01	1.10E-04	0.00E+00	1.00E+00	0.00E+00
				Pezizales	9.30E+00				
				Mucoromycotina	2.12E-09				

	Rate shift (time(short))								
	# Parameters	Likelihood	AIC	Time (Myr)	Rate modifier	Rate (non-ECM to ECM)	Rate (ECM to non-ECM)	Root (non-ECM)	Root (ECM)
**E**	**6**	-243.60	499.19	903.209	1.28E+00	8.46E-05	8.46E-05	1.00E+00	4.29E-04
				966.109	4.97E+00				
				1040.21	4.54E+00				
				1074.21	7.42E+00				
				1084.61	1.78E+00				
**N**	**6**	-245.62	503.24	903.209	1.11E+00	9.37E-05	0.00E+00	1.00E+00	0.00E+00
				966.109	3.68E+00				
				1040.21	4.61E+00				
				1074.21	5.76E+00				
				1084.61	3.03E+00				
**U**	**7**	-224.66	463.32	903.209	1.61E+00	9.47E-06	7.40E-04	9.74E-03	9.90E-01
				966.109	4.26E+00				
				1040.21	1.73E+01				
				1074.21	1.36E+01				
				1084.61	9.44E-01				

	Rate shift (time(long))								
	# Parameters	Likelihood	AIC	Time (Myr)	Rate modifier	Rate (non-ECM to ECM)	Rate (ECM to non-ECM)	Root (non-ECM)	Root (ECM)
**E**	**22**	-238.83	521.65	140.21	4.03E-05	9.69E-05	9.69E-05	1.00E+00	2.03E-13
				599.209	1.37E-06				
				654.81	1.09E-04				
				696.409	3.60E-04				
				721.01	1.55E-04				
				781.31	3.33E-04				
				841.31	2.41E+00				
				888.307	9.36E-05				
				893.01	4.26E+01				
				903.209	1.53E-04				
				938.909	1.31E-05				
				966.109	9.98E+00				
				976.709	1.07E-03				
				995.209	3.85E-05				
				1039.71	4.78E+00				
				1074.21	6.19E+00				
				1084.21	1.11E-04				
				1106.31	2.31E+00				
				1117.18	2.97E-05				
				1134.88	2.84E-06				
				1137.63	4.36E+00				
**N**	**22**	-240.29	524.58	140.21	1.93E-04	2.91E-05	0.00E+00	1.00E+00	0.00E+00
				599.209	4.15E-05				
				654.81	4.30E-04				
				696.409	2.62E-04				
				721.01	3.54E-04				
				781.31	1.03E-04				
				841.31	7.91E+00				
				888.307	1.20E-04				
				893.01	1.35E+02				
				903.209	1.61E-03				
				938.909	2.42E-04				
				966.109	3.06E+01				
				976.709	1.42E-02				
				995.209	2.71E-04				
				1039.71	1.82E+01				
				1074.21	1.81E+01				
				1084.21	8.66E-06				
				1106.31	9.50E+00				
				1117.18	9.50E-06				
				1134.88	4.16E-05				
				1137.63	1.92E+01				
**U**	**23**	-238.51	523.03	140.21	5.65E-04	1.29E-04	2.59E-04	1.00E+00	2.87E-10
				599.209	6.71E-06				
				654.81	1.63E-05				
				696.409	4.25E-05				
				721.01	2.95E-05				
				781.31	2.34E-04				
				841.31	1.84E+00				
				888.307	7.52E-05				
				893.01	3.43E+01				
				903.209	4.57E-03				
				938.909	9.50E-06				
				966.109	7.95E+00				
				976.709	9.39E-05				
				995.209	1.44E-07				
				1039.71	3.71E+00				
				1074.21	4.59E+00				
				1084.21	5.23E-05				
				1106.31	1.49E+00				
				1117.18	5.38E-05				
				1134.88	2.78E-07				
				1137.63	2.71E+00				
