# Supplementary material for: Impact of model assumptions on the inference of the evolution of ectomycorrhizal symbiosis in fungi
Source: Sci Rep. 2022 Dec 21;12:22043. doi: 10.1038/s41598-022-26514-2 (PMC9772227; doi:10.1038/s41598-022-26514-2)
Supplement: Supplementary file 5 — Supplementary Information 5. [file 41598_2022_26514_MOESM5_ESM.pdf]

Table S3: Simulation based comparison of AIC amongst all models. The table show the comparison of AIC between all models based on simulations. For each comparison the simulations are based on the model given in the column. The columns are arranged from lower to higher AIC values, based on the observed data. The cell where the model is compared with itself (grey cell) show the probability of the observed or lower ML value based on data simulated using the same model. When the model on the row is compared to a model with lower AIC (left of grey cell) it gives the probability of observing a lower AIC for the model in the column, i.e. the probability of considering the true model as better. When the model on the row is compared to a model with higher AIC (right of the grey cell) the probability of the observed, or larger, ΔAIC between the models is given, i.e. the probability of the observed difference even if the model with higher AIC is true. E=equal, N=non-reversible, U=unconstrained. Models with rate shifts are specified with either symbol or combinations of symbols where A=Agaricomycetidae, P=Pezizales, M=Mucoromycotina (except Endogonacae), T=Thelephorales, Time (5)=time with 5 geological time points, T(21)= time with all geological time points. Symbols in braces mean that that the taxa were constrained to have the same rates.

**BayesTraits**											
		**Likelihood**				**Rate (non-ECM to ECM)**	**Rate (ECM to non-ECM)**			**Root (non-ECM)**	**Root (ECM)**
**U**		-246.32				2.37E-04	3.07E-04			9.96E-01	4.24E-03
**No rate shift**											
	**# Parameters**	**Likelihood**	**AIC**			**Rate (non-ECM to ECM)**	**Rate (ECM to non-ECM)**	**CI (ECM to non-ECM**	**CI (non-ECM to ECM)**	Root (non-ECM)	Root (ECM)
**E**	**1**	-246.39	494.78			2.40E-04	2.40E-04	(1.77E-04,3.07E-04)	(1.77E-04,3.07E-04)	9.98E-01	2.43E-03
**N**	**1**	-257.45	516.90			2.61E-04	0.00E+00	(1.83E-04,4.14E-04)	(0.00E+00,0.00E+00)	1.00E+00	0.00E+00
**U**	**2**	-246.32	496.65			2.37E-04	3.07E-04	(1.57E-04,3.33E-04)	(0.00E+00,1.31E-03)	9.96E-01	4.25E-03
**Rate shift (clade: Agricomycetidae)**											
	**# Parameters**	**Likelihood**	**AIC**		**Rate modifier**	**Rate (non-ECM to ECM)**	**Rate (ECM to non-ECM)**	**CI (ECM to non-ECM**	**CI (non-ECM to ECM)**	**Root (non-ECM)**	**Root (ECM)**
**E**	**2**	-231.85	467.70		5.79E+00	1.56E-04	1.56E-04	(1.03E-04,2.15E-04)	(1.03E-04,2.15E-04)	9.99E-01	9.51E-04
**N**	**2**	-241.94	487.88		5.91E+00	1.74E-04	0.00E+00	(1.10E-04,2.81E-04)	(0.00E+00,0.00E+00)	1.00E+00	0.00E+00
**U**	**3**	-231.57	469.15		6.02E+00	1.58E-04	9.74E-05	(1.03E-04,2.32E-04)	(0.00E+00,5.53E-04)	1.00E+00	3.52E-04
**Rate shift (clade: Pezizales)**											
	**# Parameters**	**Likelihood**	**AIC**		**Rate modifier**	**Rate (non-ECM to ECM)**	**Rate (ECM to non-ECM)**	**CI (ECM to non-ECM**	**CI (non-ECM to ECM)**	**Root (non-ECM)**	**Root (ECM)**
**E**	**2**	-238.94	481.87		4.60E+00	1.93E-04	1.93E-04	(1.66E-04,2.96E-04)	(1.66E-04,2.96E-04)	9.98E-01	1.51E-03
**N**	**2**	-248.77	501.54		4.97E+00	2.11E-04	0.00E+00	(1.60E-04,4.08E-04)	(0.00E+00,0.00E+00)	1.00E+00	0.00E+00
**U**	**3**	-238.71	483.42		4.85E+00	1.95E-04	1.25E-04	(1.58E-04,3.52E-04)	(1.58E-05,6.16E-05)	9.99E-01	5.95E-04
**Rate shift (clade: Mucormycotina (except Endogonacae))**											
	**# Parameters**	**Likelihood**	**AIC**		**Rate modifier**	**Rate (non-ECM to ECM)**	**Rate (ECM to non-ECM)**	**CI (ECM to non-ECM**	**CI (non-ECM to ECM)**	**Root (non-ECM)**	**Root (ECM)**
**E**	**2**	-240.21	484.42		0.00E+00	2.72E-04	2.72E-04	(2.01E-04,3.58E-04)	(2.01E-04,3.58E-04)	9.97E-01	3.15E-03
**N**	**2**	-250.49	504.98		0.00E+00	3.00E-04	0.00E+00	(2.18E-04,1.19E-03)	(0.00E+00,0.00E+00)	1.00E+00	0.00E+00
**U**	**3**	-240.21	486.42		0.00E+00	2.72E-04	2.78E-04	(1.51E-04,4.90E-04)	(0.00E+00,9.89E-04)	9.97E-01	3.28E-03
**Rate shift (clade: Thelephorales)**											
	**# Parameters**	**Likelihood**	**AIC**		**Rate modifier**	**Rate (non-ECM to ECM)**	**Rate (ECM to non-ECM)**	**CI (ECM to non-ECM**	**CI (non-ECM to ECM)**	**Root (non-ECM)**	**Root (ECM)**
**E**	**2**	-243.93	491.87		9.29E+00	2.31E-04	2.31E-04	(1.69E-04,3.00E-04)	(1.69E-04,3.00E-04)	9.98E-01	2.23E-03
**N**	**2**	-242.63	489.26		5.55E+01	2.39E-04	0.00E+00	(1.70E-04,3.77E-04)	(0.00E+00,0.00E+00)	1.00E+00	0.00E+00
**U**	**3**	-242.35	490.70		6.95E+01	2.38E-04	3.43E-05	(1.61E-04,3.38E-04)	(0.00E+00,1.61E-04)	1.00E+00	4.27E-05
**Rate shift (Agricomycetidae, Thelephorales)**											
	**# Parameters**	**Likelihood**	**AIC**	**Clade**	**Rate modifier**	**Rate (non-ECM to ECM)**	**Rate (ECM to non-ECM)**	**CI (ECM to non-ECM**	**CI (non-ECM to ECM)**	**Root (non-ECM)**	**Root (ECM)**
**E**	**3**	-228.59	463.18	Agricomycetidae	6.21E+00	1.45E-04	1.45E-04	(9.35E-05,2.05E-04)	(9.35E-05,2.05E-04)	9.99E-01	8.19E-04
				Thelephorale	1.48E+01						
**N**	**3**	-225.31	456.61	Agricomycetidae	6.85E+00	1.50E-04	0.00E+00	(9.17E-05,2.40E-04)	(0.00E+00,0.00E+00)	1.00E+00	0.00E+00
				Thelephorale	8.84E+01						
**U**	**4**	-225.24	458.47	Agricomycetidae	6.82E+00	1.50E-04	7.49E-06	(8.83E-05,2.26E-04)	(0.00E+00,9.25E-05)	1.00E+00	1.98E-06
				Thelephorale	9.59E+01						
**Rate shift (Agricomycetidae, Mucormycotina (except Endogonacae))**											
	**# Parameters**	**Likelihood**	**AIC**	**Clade**	**Rate modifier**	**Rate (non-ECM to ECM)**	**Rate (ECM to non-ECM)**	**CI (ECM to non-ECM**	**CI (non-ECM to ECM)**	**Root (non-ECM)**	**Root (ECM)**
**E**	**3**	-227.75	461.50	Agricomycetidae	5.00E+00	1.81E-04	1.81E-04	(1.19E-04,2.49E-04)	(1.19E-04,2.49E-04)	9.99E-01	1.29E-03
				Mucoromycotina	0.00E+00						
**N**	**3**	-237.26	480.52	Agricomycetidae	5.06E+00	2.03E-04	0.00E+00	(1.35E-04,7.06E-04)	(0.00E+00,0.00E+00)	1.00E+00	0.00E+00
				Mucoromycotina	0.00E+00						
**U**	**4**	-227.38	462.76	Agricomycetidae	5.21E+00	1.84E-04	1.05E-04	(1.07E-04,3.70E-04)	(0.00E+00,4.85E-04)	1.00E+00	4.11E-04
				Mucoromycotina	0.00E+00						
**Rate shift (Agaricomycetidae, Pezizales)**											
	**# Parameters**	**Likelihood**	**AIC**	**Clade**	**Rate modifier**	**Rate (non-ECM to ECM)**	**Rate (ECM to non-ECM)**	**CI (ECM to non-ECM**	**CI (non-ECM to ECM)**	**Root (non-ECM)**	**Root (ECM)**
**E**	**3**	-219.21	444.43	Agricomycetidae	9.37E+00	9.64E-05	9.64E-05	(1.07E-04,3.70E-04)	(0.00E+00,4.85E-04)	1.00E+00	3.44E-04
				Pezizales	9.21E+00						
**N**	**3**	-228.23	462.46	Agricomycetidae	9.21E+00	1.12E-04	0.00E+00	(6.12E-05,1.83E-04)	(0.00E+00,0.00E+00)	1.00E+00	0.00E+00
				Pezizales	9.38E+00						
**U**	**4**	-217.87	443.74	Agricomycetidae	1.01E+01	9.79E-05	3.35E-05	(5.30E-05,1.51E-04)	(0.00E+00,1.68E-04)	1.00E+00	3.96E-05
				Pezizales	1.01E+01						
**Rate shift (Pezizales, Mucormycotina (except Endogonacae))**											
	**# Parameters**	**Likelihood**	**AIC**	**Clade**	**Rate modifier**	**Rate (non-ECM to ECM)**	**Rate (ECM to non-ECM)**	**CI (ECM to non-ECM**	**CI (non-ECM to ECM)**	**Root (non-ECM)**	**Root (ECM)**
**E**	**3**	-233.91	473.81	Pezizales	4.00E+00	2.22E-04	2.22E-04	(1.54E-04,2.98E-04)	(1.54E-04,2.98E-04)	9.98E-01	2.01E-03
				Mucoromycotina	0.00E+00						
**N**	**3**	-243.12	492.24	Pezizales	4.28E+00	2.45E-04	0.00E+00	(1.68E-04,8.84E-04)	(0.00E+00,0.00E+00)	1.00E+00	0.00E+00
				Mucoromycotina	0.00E+00						
**U**	**4**	-233.57	475.14	Pezizales	4.24E+00	2.25E-04	1.32E-04	(1.34E-04,3.50E-04)	(0.00E+00,7.13E-04)	9.99E-01	6.64E-04
				Mucoromycotina	5.28E-10						
**Rate shift (Pezizales, Thelephorales)**											
	**# Parameters**	**Likelihood**	**AIC**	**Clade**	**Rate modifier**	**Rate (non-ECM to ECM)**	**Rate (ECM to non-ECM)**	**CI (ECM to non-ECM**	**CI (non-ECM to ECM)**	**Root (non-ECM)**	**Root (ECM)**
**E**	**3**	-236.08	478.16	Pezizales	4.85E+00	1.83E-04	1.83E-04	(1.25E-04,2.50E-04)	(1.25E-04,2.50E-04)	9.99E-01	1.35E-03
				Thelephorales	1.17E+01						
**N**	**3**	-233.00	472.00	Pezizales	5.58E+00	1.88E-04	0.00E+00	(1.24E-04,2.99E-04)	(0.00E+00,0.00E+00)	1.00E+00	0.00E+00
				Thelephorales	7.06E+01						
**U**	**4**	-232.91	473.81	Pezizales	5.54E+00	1.88E-04	1.13E-05	(1.25E-04,2.71E-04)	(0.00E+00,8.61E-05)	1.00E+00	4.51E-06
				Thelephorales	7.78E+01						
**Rate shift (Mucormycotina (except Endogonacae), Thelephorales)**											
	**# Parameters**	**Likelihood**	**AIC**	**Clade**	**Rate modifier**	**Rate (non-ECM to ECM)**	**Rate (ECM to non-ECM)**	**CI (ECM to non-ECM**	**CI (non-ECM to ECM)**	**Root (non-ECM)**	**Root (ECM)**
**E**	**3**	-237.98	481.96	Mucoromycotina	0.00E+00	2.62E-04	2.62E-04	(1.91E-04,3.42E-04)	(1.91E-04,3.42E-04)	9.97E-01	2.90E-03
				Thelephorales	8.14E+00						
**N**	**3**	-236.25	478.50	Mucoromycotina	0.00E+00	2.75E-04	0.00E+00	(1.96E-04,1.19E-03)	(0.00E+00,0.00E+00)	1.00E+00	0.00E+00
				Thelephorales	4.83E+01						
**U**	**4**	-236.03	480.06	Mucoromycotina	0.00E+00			(1.80E-04,7.72E-04)	(0.00E+00,1.78E-04)		
				Thelephorales	5.87E+01	2.73E-04	3.29E-05			1.00E+00	3.97E-05
**Rate shift (Agricomycetidae, Pezizales, Thelephorales;** Agricomycetidae, Pezizales=same rates**)**											
	**# Parameters**	**Likelihood**	**AIC**	**Clade**	**Rate modifier**	**Rate (non-ECM to ECM)**	**Rate (ECM to non-ECM)**	**CI (ECM to non-ECM**	**CI (non-ECM to ECM)**	**Root (non-ECM)**	**Root (ECM)**
**E**	3	-215.00	436.00	Agricomycetidae	1.06E+01	8.48E-05	8.48E-05	(4.17E-05,1.33E-04)	(4.17E-05,1.33E-04)	1.00E+00	2.63E-04
				Pezizales	1.06E+01						
				Thelephorales	2.56E+01						
**N**	3	-209.57	425.15	Agricomycetidae	1.20E+01	8.64E-05	0.00E+00	(4.19E-05,1.45E-04)	(0.00E+00,0.00E+00)	1.00E+00	0.00E+00
				Pezizales	1.20E+01						
				Thelephorales	1.54E+02						
**U**	4	-209.57	427.13	Agricomycetidae	1.20E+01	8.64E-05	9.75E-07	(4.73E-05,1.52E-04)	(0.00E+00,4.24E-05)	1.00E+00	3.29E-08
				Pezizales	1.20E+01						
				Thelephorales	1.56E+02						
**Rate shift (Agricomycetidae, Mucormycotina (except Endogonacae), Thelephorales)**											
	**# Parameters**	**Likelihood**	**AIC**	**Clade**	**Rate modifier**	**Rate (non-ECM to ECM)**	**Rate (ECM to non-ECM)**	**CI (ECM to non-ECM**	**CI (non-ECM to ECM)**	**Root (non-ECM)**	**Root (ECM)**
**E**	4	-224.76	457.53	Agricomycetidae	5.36E+00	1.69E-04	1.69E-04	(1.07E-04,2.33E-04)	(1.07E-04,2.33E-04)	9.99E-01	1.11E-03
				Mucoromycotina	0.00E+00						
				Thelephorales	1.28E+01						
**N**	4	-221.27	450.54	Agricomycetidae	5.87E+00	1.75E-04	0.00E+00	(1.12E-04,3.49E-04)	(0.00E+00,0.00E+00)	1.00E+00	0.00E+00
				Mucoromycotina	0.00E+00						
				Thelephorales	7.57E+01						
**U**	5	-221.21	452.42	Agricomycetidae	5.85E+00	1.75E-04	7.75E-06	(1.06E-04,5.11E-04)	(0.00E+00,8.00E-05)	1.00E+00	2.13E-06
				Mucoromycotina	0.00E+00						
				Thelephorales	8.14E+01						
**Rate shift (Pezizales, Mucormycotina (except Endogonacae), Thelephorales)**											
	**# Parameters**	**Likelihood**	**AIC**	**Clade**	**Rate modifier**	**Rate (non-ECM to ECM)**	**Rate (ECM to non-ECM)**	**CI (ECM to non-ECM**	**CI (non-ECM to ECM)**	**Root (non-ECM)**	**Root (ECM)**
**E**	4	-231.30	470.60	Pezizales	4.22E+00	2.11E-04	2.11E-04	(1.49E-04,2.88E-04)	(1.49E-04,2.88E-04)	9.98E-01	1.79E-03
				Mucoromycotina	0.00E+00						
				Thelephorales	1.02E+01						
**N**	4	-227.97	463.93	Pezizales	4.81E+00	2.18E-04	0.00E+00	(1.47E-04,8.77E-04)	(0.00E+00,0.00E+00)	1.00E+00	0.00E+00
				Mucoromycotina	3.24E-09						
				Thelephorales	6.09E+01						
**U**	5	-227.89	465.77	Pezizales	4.78E+00	2.17E-04	1.13E-05	(1.46E-04,5.49E-04)	(0.00E+00,8.30E-05)	1.00E+00	4.60E-06
				Mucoromycotina	4.00E-10						
				Thelephorales	6.63E+01						
**Rate shift (Agricomycetidae, Pezizales, Thelephorales)**											
	**# Parameters**	**Likelihood**	**AIC**	**Clade**	**Rate modifier**	**Rate (non-ECM to ECM)**	**Rate (ECM to non-ECM)**	**CI (ECM to non-ECM**	**CI (non-ECM to ECM)**	**Root (non-ECM)**	**Root (ECM)**
**E**	4	-215.00	438.00	Agricomycetidae	1.07E+01	8.48E-05	8.48E-05	(4.16E-05,1.28E-04)	(4.16E-05,1.28E-04)	1.00E+00	2.63E-04
				Pezizales	1.05E+01						
				Thelephorales	2.56E+01						
**N**	4	-209.57	427.14	Agricomycetidae	1.19E+01	8.64E-05	0.00E+00	(4.18E-05,1.40E-04)	(0.00E+00,0.00E+00)	1.00E+00	0.00E+00
				Pezizales	1.21E+01						
				Thelephorales	1.54E+02						
**U**	5	-209.57	429.13	Agricomycetidae	1.19E+01	8.64E-05	9.73E-07	(4.37E-05,1.50E-04)	(0.00E+00,2.91E-05)	1.00E+00	3.28E-08
				Pezizales	1.21E+01						
				Thelephorales	1.56E+02						
**Rate shift (Agricomycetidae, Pezizales, Mucormycotina (except Endogonacae))**											
	**# Parameters**	**Likelihood**	**AIC**	**Clade**	**Rate modifier**	**Rate (non-ECM to ECM)**	**Rate (ECM to non-ECM)**	**CI (ECM to non-ECM**	**CI (non-ECM to ECM)**	**Root (non-ECM)**	**Root (ECM)**
**E**	**4**	-216.65	441.29	Agricomycetidae	7.98E+00	1.13E-04	1.13E-04	(6.22E-05,1.72E-04)	(6.22E-05,1.72E-04)	1.00E+00	4.79E-04
				Pezizales	7.84E+00						
				Mucoromycotina	0.00E+00						
**N**	**4**	-225.21	458.42	Agricomycetidae	7.79E+00	1.32E-04	0.00E+00	(7.40E-05,3.49E-04)	(0.00E+00,0.00E+00)	1.00E+00	0.00E+00
				Pezizales	7.93E+00						
				Mucoromycotina	0.00E+00						
**U**	**5**	-215.24	440.47	Agricomycetidae	8.54E+00	1.15E-04	3.86E-05	(6.20E-05,2.29E-04)	(0.00E+00,2.05E-04)	1.00E+00	5.26E-05
				Pezizales	8.61E+00						
				Mucoromycotina	0.00E+00						
**Rate shift (Agricomycetidae, Pezizales, Mucormycotina (except Endogonacae), Thelephorales;** Agricomycetidae, Pezizales=same rates**))**											
	**# Parameters**	**Likelihood**	**AIC**	**Clade**	**Rate modifier**	**Rate (non-ECM to ECM)**	**Rate (ECM to non-ECM)**	**CI (ECM to non-ECM**	**CI (non-ECM to ECM)**	**Root (non-ECM)**	**Root (ECM)**
**E**	4	-212.74	433.48	Agricomycetidae	9.01E+00	9.97E-05	9.97E-05	(4.92E-05,1.56E-04)	(4.92E-05,1.56E-04)	1.00E+00	3.67E-04
				Pezizales	9.01E+00						
				Mucoromycotina	7.69E-10						
				Thelephorales	2.17E+01						
**N**	4	-207.24	422.47	Agricomycetidae	1.01E+01	1.02E-04	0.00E+00	(5.16E-05,3.49E-04)	(0.00E+00,0.00E+00)	1.00E+00	0.00E+00
				Pezizales	1.01E+01						
				Mucoromycotina	1.29E-16						
				Thelephorales	1.30E+02						
**U**	5	-207.23	424.46	Agricomycetidae	1.01E+01	1.02E-04	1.07E-06	(5.55E-05,3.49E-04)	(0.00E+00,4.17E-05)	1.00E+00	3.95E-08
				Pezizales	1.01E+01						
				Mucoromycotina	0.00E+00						
				Thelephorales	1.32E+02						
**Rate shift (Agricomycetidae, Pezizales, Mucormycotina (except Endogonacae), Thelephorales)**											
	**# Parameters**	**Likelihood**	**AIC**	**Clade**	**Rate modifier**	**Rate (non-ECM to ECM)**	**Rate (ECM to non-ECM)**	**CI (ECM to non-ECM**	**CI (non-ECM to ECM)**	**Root (non-ECM)**	**Root (ECM)**
**E**	**5**	-212.74	435.47	Agricomycetidae	9.06E+00	9.97E-05	9.97E-05	(5.56E-05,1.55E-04)	(5.56E-05,1.55E-04)	1.00E+00	3.67E-04
				Pezizales	8.91E+00						
				Mucoromycotina	3.32E-09						
				Thelephorales	2.17E+01						
**N**	**5**	-207.23	424.47	Agricomycetidae	1.01E+01	1.02E-04	0.00E+00	(5.54E-05,3.49E-04)	(0.00E+00,0.00E+00)	1.00E+00	0.00E+00
				Pezizales	1.03E+01						
				Mucoromycotina	1.81E-07						
				Thelephorales	1.30E+02						
**U**	**6**	-207.23	426.47	Agricomycetidae	1.01E+01	1.02E-04	0.00E+00	(4.88E-05,3.49E-04)	(0.00E+00,0.00E+00)	1.00E+00	0.00E+00
				Pezizales	1.03E+01						
				Mucoromycotina	0.00E+00						
				Thelephorales	1.30E+02						
**Rate shift (time(short))**											
	**# Parameters**	**Likelihood**	**AIC**	**Time (Myr)**	**Rate modifier**	**Rate (non-ECM to ECM)**	**Rate (ECM to non-ECM)**	**CI (ECM to non-ECM**	**CI (non-ECM to ECM)**	**Root (non-ECM)**	**Root (ECM)**
**E**	**6**	-233.81	479.61	903.209	2.77E+00	3.93E-05	3.93E-05	(1.43E-09,1.03E-04)	(1.43E-09,1.03E-04)	1.00E+00	4.67E-04
				966.109	1.11E+01						
				1040.21	1.21E+01						
				1074.21	1.35E+01						
				1084.61	9.20E+00						
**N**	**6**	-244.69	501.38	903.209	2.29E+00	4.33E-05	0.00E+00	(1.82E-09,1.29E-04)	(0.00E+00,0.00E+00)	1.00E+00	0.00E+00
				966.109	8.57E+00						
				1040.21	1.05E+01						
				1074.21	1.50E+01						
				1084.61	8.27E+00						
**U**	**7**	-211.53	437.07	903.209	2.72E+00	5.13E-06	4.32E-04	(7.45E-12,2.20E-05)	(1.76E-10,1.56E-03)	1.06E-02	9.89E-01
				966.109	9.56E+00						
				1040.21	2.76E+01						
				1074.21	2.06E+01						
				1084.61	2.91E+00						
**Rate shift (time(long))**											
	**# Parameters**	**Likelihood**	**AIC**	**Time (Myr)**	**Rate modifier**	**Rate (non-ECM to ECM)**	**Rate (ECM to non-ECM)**	**CI (ECM to non-ECM**	**CI (non-ECM to ECM)**	**Root (non-ECM)**	**Root (ECM)**
**E**	**22**	-228.29	500.58	140.21	2.97E-02	3.33E-06	3.33E-06	(2.76E-09,1.92E-05)	(2.76E-09,1.92E-05)	1.00E+00	1.63E-10
				599.209	4.74E-03						
				654.81	2.12E-02						
				696.409	1.30E-02						
				721.01	8.50E-03						
				781.31	8.33E-03						
				841.31	6.71E+01						
				888.307	6.04E-04						
				893.01	1.25E+03						
				903.209	4.11E-01						
				938.909	1.08E-03						
				966.109	3.10E+02						
				976.709	8.69E-03						
				995.209	1.16E-03						
				1039.71	1.73E+02						
				1074.21	1.59E+02						
				1084.21	5.64E-05						
				1106.31	7.34E+01						
				1117.18	8.50E-03						
				1134.88	5.14E-04						
				1137.63	1.45E-04						
**N**	**22**	-238.65	521.30	140.21	3.54E-03	5.52E-06	0.00E+00	(4.45E-09,2.13E-05)	(0.00E+00,0.00E+00)	1.00E+00	0.00E+00
				599.209	4.50E-03						
				654.81	1.05E-03						
				696.409	4.97E-04						
				721.01	1.37E-04						
				781.31	8.78E-03						
				841.31	3.80E+01						
				888.307	5.91E-04						
				893.01	7.01E+02						
				903.209	1.34E+00						
				938.909	6.75E-03						
				966.109	1.69E+02						
				976.709	2.02E-03						
				995.209	1.89E-03						
				1039.71	1.01E+02						
				1074.21	1.16E+02						
				1084.21	1.09E-03						
				1106.31	4.75E+01						
				1117.18	1.55E-03						
				1134.88	5.56E-05						
				1137.63	7.37E-04						
**U**	**23**	-206.73	459.46	140.21	1.22E+03	3.39E-08	3.10E-06	(2.66E-11,2.04E-06)	(6.30E-10,1.59E-04)	7.69E-03	9.92E-01
				599.209	2.40E+00						
				654.81	6.13E+02						
				696.409	5.21E+01						
				721.01	1.30E+01						
				781.31	1.60E+03						
				841.31	6.23E+01						
				888.307	2.50E+00						
				893.01	1.05E+01						
				903.209	1.93E+02						
				938.909	1.92E+03						
				966.109	8.72E+02						
				976.709	7.68E-02						
				995.209	4.32E+03						
				1039.71	4.55E+03						
				1074.21	2.49E+03						
				1084.21	3.34E+01						
				1106.31	8.06E+02						
				1117.18	1.20E+00						
				1134.88	2.22E+00						
				1137.63	2.56E+00						
