# Supplementary material for: Impact of model assumptions on the inference of the evolution of ectomycorrhizal symbiosis in fungi
Source: Sci Rep. 2022 Dec 21;12:22043. doi: 10.1038/s41598-022-26514-2 (PMC9772227; doi:10.1038/s41598-022-26514-2)
Supplement: Supplementary file 6 — Supplementary Information 6. [file 41598_2022_26514_MOESM6_ESM.pdf]

Table S4: Ancestral state reconstruction using equal, non-reversal and unconstrained models with and without rate shifts. The “likelihood” column gives the likelihood value for a certain model (specified at the top of each section in dark grey) given the rate of change from non-ectomycorrhizal to ectomycorrhizal and vice versa (“Rate”). The “AIC” column gives the AIC score for each model based on the likelihood and number of parameters written in “#Parameter” column. Root (ECM/non-ECM) is the probability of having an ectomycorrhizal respectively non-ectomycorrhizal root. For models with rate shift in clade, the column with “Clades” specifies the clades being tested and the column with “Rate modifier” gives the value for the rate of evolution of each clade under the specified model. For models with rate shift in time, the column with “Time” shows the time points used for the analyses. E=equal, N=non-reversible, U=unconstrained models. The lowest AIC value for each of the tree models under different models of rate shifts is marked in green, and the overall lowest AIC in darker green. The top row gives the “Rate” values obtained using BayesTraits that were used as starting points for the analyses with no rate shift with phylommand-treeator. All the other analyses were conducted using phylommand-treeator.

				(A+P)+M+T	(A+P)+M+T	A+P+M+T	(A+P)+T	A+P+M+T	(A+P)+T	A+P+T	A+P+T	(A+P)+M+T	A+P+M+T	(A+P)+T	Time (5)	A+P+T
			AIC	422.47	424.46	424.468	425.146	426.468	427.132	427.142	429.13	433.476	435.474	436.002	437.066	438
				N	U	N	N	U	U	N	U	E	E	E	U	E
NO RATE SHIFT	E, N, U	E	494.778	0.999	0.993	1	0.296	0.97	0.951	0.997	0.99	1	1	1	0.866	1
		N	516.904	0.968	0.978	0.963	0.531	0.962	0.904	0.97	0.981	1	1	1	0.882	1
		U	496.646	0.998	0.977	0.963	0.381	0.968	0.921	0.97	0.965	1	1	1	0.799	1
RATE SHIFT (CLADE)	A	E	467.698	0.999	0.662	1	0.359	0.674	0.902	0.997	0.684	1	1	0.997	0.566	0.999
		N	487.876	0.998	0.995	0.963	0.595	0.97	0.934	0.97	0.991	1	1	0.998	0.883	1
		U	469.148	0.999	0.999	1	0.43	0.97	0.884	0.97	0.981	1	1	0.996	0.912	1
	P	E	481.87	0.999	0.725	1	0.31	0.721	0.876	0.996	0.743	1	1	1	0.567	1
		N	501.54	0.982	0.995	0.929	0.554	0.998	0.952	0.932	0.985	1	1	1	0.88	1
		U	483.42	0.999	0.993	0.987	0.388	0.938	0.856	0.932	0.954	1	1	1	0.914	1
	M	E	484.42	0.999	0.731	1	0.185	0.741	0.928	0.997	0.776	1	1	1	0.566	1
		N	504.984	0.959	0.996	0.95	0.409	0.97	0.942	0.97	0.989	1	1	1	0.844	1
		U	486.418	0.959	0.97	0.95	0.254	0.959	0.899	0.97	0.979	0.999	1	1	0.9	1
	T	E	491.866	0.999	0.724	1	0.35	0.74	0.918	0.997	0.743	1	1	1	0.567	1
		N	489.258	0.998	0.998	0.963	0.472	0.999	0.957	0.97	0.989	1	1	1	0.893	1
		U	490.698	0.999	0.999	1	0.469	0.97	0.911	0.97	0.981	1	1	1	0.922	1
	A+T	E	463.184	0.999	0.607	1	0.42	0.622	0.857	0.999	0.624	0.999	1	0.99	0.566	0.999
		N	456.612	0.998	0.997	0.994	0.508	1	0.92	0.941	0.992	0.999	1	0.995	0.904	1
		U	458.472	0.999	0.995	1	0.509	0.996	0.824	0.95	0.96	0.999	0.998	0.991	0.925	1
	P+T	E	478.162	0.999	0.685	1	0.374	0.696	0.865	1	0.691	0.999	1	1	0.567	1
		N	471.998	0.999	0.997	0.987	0.479	0.999	0.954	0.931	0.99	0.999	1	1	0.908	1
		U	473.81	0.999	0.999	0.999	0.487	0.989	0.843	0.931	0.954	1	1	1	0.93	0.999
	M+T	E	481.956	0.999	0.703	1	0.225	0.714	0.915	1	0.74	1	1	1	0.567	1
		N	478.5	0.959	0.999	0.95	0.309	1	0.941	0.97	0.99	1	1	1	0.88	1
		U	480.062	0.961	0.97	0.95	0.317	0.96	0.882	0.97	0.979	1	1	1	0.919	1
	A+M	E	461.504	0.999	0.637	1	0.223	0.654	0.899	1	0.684	0.998	1	0.997	0.567	1
		N	480.52	0.959	0.999	0.95	0.487	1	0.938	0.97	0.992	0.996	0.999	0.998	0.866	1
		U	462.758	0.961	0.969	0.95	0.301	0.96	0.887	0.97	0.979	0.997	1	0.997	0.91	1
	P+M	E	473.81	0.999	0.701	1	0.202	0.702	0.872	1	0.739	0.999	1	1	0.567	1
		N	492.24	0.903	0.998	0.903	0.444	0.999	0.942	0.932	0.987	0.999	1	1	0.86	1
		U	475.142	0.907	0.938	0.903	0.276	0.923	0.848	0.932	0.952	0.998	0.999	0.998	0.906	1
	A+P	E	444.428	0.999	0.576	0.999	0.353	0.582	0.829	0.996	0.577	0.977	0.974	0.911	0.567	0.899
		N	462.46	0.995	0.995	0.987	0.611	0.999	0.9	0.926	0.981	0.982	0.98	0.966	0.897	0.952
		U	443.742	0.998	0.998	0.999	0.459	0.987	0.813	0.93	0.945	0.975	0.976	0.941	0.919	0.906
	(A+P)+T	E	436.002	0.988	0.5	0.99	0.322	0.494	0.191	0.975	0.474	0.992	0.823	0.908	0.433	0.026
		N	425.146	0.979	0.993	0.845	0.978	0.011	0.107	0.127	0.001	0.002	0.003	0.033	0.002	0
		U	427.132	0.984	0.978	0.97	0.222	0.809	0.94	0.115	0.082	0.003	0.004	0.001	0.002	0.001
	A+P+T	E	438	0.993	0.988	0.992	0.421	0.989	0.888	0.987	0.962	0.963	0.991	0.829	0.913	0.978
		N	427.142	0.957	0.873	0.979	0.85	0.55	0.874	0.91	0.71	0.003	0.003	0.002	0.002	0
		U	429.13	0.972	0.945	0.988	0.806	0.935	0.701	0.948	0.94	0.007	0.005	0.006	0.002	0.001
	A+M+T	E	457.528	1	0.996	1	0.285	1	0.933	1	0.995	0.995	1	0.992	0.89	1
		N	450.542	0.935	0.929	0.917	0.352	0.928	0.805	0.941	0.954	0.996	0.997	0.995	0.906	1
		U	452.424	0.998	0.938	0.927	0.359	0.934	0.851	0.984	0.946	0.995	0.997	0.991	0.88	1
	P+M+T	E	470.602	0.999	0.996	1	0.246	0.999	0.938	1	0.995	0.999	1	1	0.892	1
		N	463.93	0.906	0.936	0.903	0.341	0.921	0.825	0.93	0.952	0.999	1	1	0.902	1
		U	465.774	1	0.925	0.904	0.347	0.923	0.876	0.977	0.935	0.999	0.999	0.999	0.89	0.999
	A+P+M	E	441.29	0.999	0.992	0.994	0.239	0.996	0.907	0.995	0.989	0.904	0.892	0.94	0.879	0.908
		N	458.42	0.899	0.929	0.898	0.526	0.914	0.807	0.929	0.937	0.962	0.943	0.976	0.897	0.965
		U	440.472	0.996	0.917	0.903	0.359	0.913	0.846	0.976	0.917	0.925	0.9	0.947	0.86	0.935
	(A+P)+M+T	E	433.476	0.981	0.962	0.973	0.187	0.95	0.886	0.975	0.966	0.85	0.031	0.033	0.044	0.038
		N	422.47	0.936	0.703	0.148	0.053	0.118	0.155	0.032	0.034	0.001	0.003	0	0.002	0
		U	424.46	0.955	0.908	0.104	0.068	0.117	0.216	0.046	0.039	0.002	0.004	0	0.002	0.002
	A+P+M+T	E	435.474	0.982	0.976	0.984	0.24	0.964	0.889	0.986	0.983	0.841	0.864	0.123	0.059	0.046
		N	424.468	0.859	0.225	0.9	0.119	0.977	0.228	0.056	0.05	0.003	0.003	0.001	0.003	0
		U	426.468	0.947	0.876	0.997	0.118	0.886	0.212	0.053	0.049	0.003	0.003	0.001	0.003	0
RATE SHIFT (TIME)	Time (5)	E	479.614	1	1	1	0.104	0.999	0.958	1	0.997	1	1	1	0.913	1
		N	501.382	1	1	1	0.309	0.999	0.93	0.992	0.984	1	1	1	0.908	1
		U	437.066	1	1	1	0.03	0.999	0.953	0.998	0.994	1	1	1	0.856	0
	Time (21)	E	500.576	0.834	0.812	1	0.088	1	0.914	1	1	0.895	1	1	0.993	1
		N	521.298	0.833	0.83	1	0.254	1	0.939	1	1	0.948	1	1	0.993	1
		U	459.458	0.822	0.804	0.999	0.031	0.997	0.906	0.996	0.997	0.89	0.995	0.995	0.988	0.996

			A+P+M	A+P+M	A+P	A+P	A+M+T	A+M+T	A+T	A+M+T	A+P+M	A+T	Time (21)	A+M	A+P	A+M	A+T	P+M+T
		AIC	440.472	441.29	443.742	444.428	450.542	452.424	456.612	457.528	458.42	458.472	459.458	461.504	462.46	462.758	463.184	463.93
			U	E	U	E	N	U	N	E	N	U	U	E	N	U	E	N
E, N, U	E	494.778	1	1	0.761	1	0.999	0.972	0.997	1	1	0.822	0.284	1	0.998	0.529	1	0.998
	N	516.904	1	1	0.996	1	0.945	0.994	0.885	1	0.971	0.975	0.326	1	0.955	0.963	1	0.951
	U	496.646	1	1	0.76	1	0.999	0.972	0.885	1	1	0.801	0.119	1	0.955	0.532	0.999	0.998
A	E	467.698	0.658	1	0.512	0.998	0.999	0.576	0.996	0.999	0.999	0.513	0.616	1	1	0.324	0.76	0.998
	N	487.876	0.996	1	0.601	0.998	0.999	0.97	0.874	0.998	0.993	0.774	0.275	0.991	0.92	0.351	0.932	0.998
	U	469.148	1	0.999	0.991	0.997	0.999	1	0.886	0.989	0.998	0.918	0.332	0.983	0.929	0.956	0.896	0.998
P	E	481.87	0.701	1	0.557	1	0.998	0.666	0.999	1	1	0.616	0.617	1	1	0.426	1	0.998
	N	501.54	0.999	1	0.715	1	0.991	0.975	0.885	1	0.997	0.822	0.276	1	0.91	0.539	1	0.998
	U	483.42	1	1	0.985	1	0.994	1	0.887	1	1	0.968	0.326	1	0.911	0.958	0.999	0.998
M	E	484.42	0.709	1	0.601	1	0.999	0.641	0.998	1	1	0.618	0.615	0.999	1	0.395	1	0.998
	N	504.984	0.999	1	0.756	1	0.857	0.971	0.885	1	0.955	0.823	0.2	0.997	0.954	0.487	1	0.909
	U	486.418	1	0.999	0.947	1	0.857	0.985	0.885	0.997	0.956	0.922	0.274	0.994	0.954	0.953	0.998	0.91
T	E	491.866	0.743	1	0.612	1	0.999	0.634	0.999	1	1	0.59	0.615	1	1	0.426	1	0.998
	N	489.258	1	1	0.762	1	0.998	0.977	0.884	1	1	0.814	0.288	1	0.955	0.542	1	0.995
	U	490.698	1	1	0.998	1	0.999	1	0.886	1	1	0.964	0.341	1	0.957	0.964	0.998	0.994
A+T	E	463.184	0.661	1	0.516	1	0.999	0.531	0.983	1	0.999	0.462	0.616	0.997	1	0.329	0.856	0.001
	N	456.612	0.998	1	0.612	0.998	0.99	0.993	0.95	0.003	0	0.284	0.692	0.001	0.001	0.58	0.01	0
	U	458.472	1	1	0.988	0.999	0.996	0.984	0.961	0.98	1	0.984	0.608	0.003	0.001	0.008	0.024	0
P+T	E	478.162	0.705	1	0.562	1	0.999	0.636	1	1	1	0.588	0.618	1	1	0.43	1	0.998
	N	471.998	1	1	0.723	1	0.994	0.995	0.886	1	1	0.815	0.291	1	0.91	0.544	1	0.987
	U	473.81	1	1	0.981	1	0.998	1	0.993	1	1	0.952	0.335	1	1	0.958	0.999	0.995
M+T	E	481.956	0.712	1	0.608	1	0.999	0.604	0.999	1	1	0.585	0.616	1	1	0.397	1	0.997
	N	478.5	1	1	0.759	1	0.852	0.993	0.883	1	0.955	0.815	0.203	0.997	0.954	0.495	1	0.848
	U	480.062	1	1	0.947	1	0.858	0.985	0.968	0.996	0.956	0.904	0.283	0.997	0.987	0.955	0.998	0.88
A+M	E	461.504	0.633	0.999	0.511	0.999	0.999	0.557	0.997	0.705	0.999	0.515	0.616	0.86	0	0.696	0.043	0
	N	480.52	0.992	0.999	0.601	0.998	0.845	0.965	0.88	0.935	0.901	0.776	0.198	0.818	0.924	0.128	0.943	0.91
	U	462.758	1	0.995	0.925	0.997	0.857	0.975	0.964	0.883	0.914	0.9	0.28	0.81	0.981	0.496	0.082	0
P+M	E	473.81	0.679	1	0.558	1	0.998	0.643	0.999	1	1	0.614	0.616	1	1	0.393	0.999	0.998
	N	492.24	0.989	1	0.715	1	0.857	0.983	0.885	1	0.896	0.822	0.195	0.999	0.909	0.489	1	0.812
	U	475.142	1	0.996	0.929	0.999	0.859	0.985	0.96	0.996	0.899	0.914	0.27	0.995	0.984	0.914	0.997	0.825
A+P	E	444.428	0.568	0.996	0.404	0.914	0	0.395	0	0	0	0.43	0.377	0	0	0.609	0	0
	N	462.46	0.927	0.965	0.209	0.852	0.993	0.986	0.877	0.998	0.98	0.779	0.271	0.988	0.984	0.64	0.045	0.002
	U	443.742	0.996	0.951	0.616	0.134	0	0	0	0	0	0.014	0.512	0	0	0.013	0	0
(A+P)+T	E	436.002	0.413	0.003	0.566	0	0	0.392	0	0	0	0.419	0.37	0	0	0.558	0	0
	N	425.146	0	0.001	0.429	0	0	0	0	0	0	0.156	0.376	0	0	0.401	0	0
	U	427.132	0	0.001	0	0	0	0	0	0	0	0.009	0.305	0	0	0.007	0	0
A+P+T	E	438	0.004	0.004	0.558	0.001	0	0	0	0	0	0.19	0.546	0	0	0.477	0	0
	N	427.142	0	0.001	0.005	0	0	0	0	0	0	0.013	0.322	0	0	0.011	0	0
	U	429.13	0	0.001	0.475	0	0	0	0	0	0	0.189	0.39	0	0	0.44	0	0
A+M+T	E	457.528	0.996	1	0.613	1	0.973	0.945	0.986	0.892	0	0.281	0.784	0.005	0	0.644	0.011	0
	N	450.542	1	0.998	0.917	0.998	0.976	0.493	0.013	0.003	0.001	0.1	0.634	0	0	0.008	0.001	0
	U	452.424	0.997	0.995	0.61	0.999	0.95	0.974	0.019	0.011	0.001	0.269	0.82	0.002	0	0.59	0.004	0
P+M+T	E	470.602	0.993	1	0.72	1	1	0.997	1	0.999	1	0.817	0.198	1	1	0.498	1	0.977
	N	463.93	1	1	0.935	1	0.857	0.983	0.959	1	0.896	0.899	0.268	0.999	0.983	0.917	1	0.704
	U	465.774	0.999	0.998	0.714	0.999	0.986	0.956	0.982	0.998	1	0.8	0.139	0.997	0.991	0.498	0.997	0.957
A+P+M	E	441.29	0.537	0.876	0.636	0.027	0	0	0	0	0	0.183	0.669	0	0	0.534	0	0
	N	458.42	0.596	0.84	0	0.902	0.854	0.969	0.95	0.948	0.95	0.122	0.672	0.041	0.03	0.13	0.011	0
	U	440.472	0.966	0.114	0.624	0.032	0	0	0	0	0	0.188	0.728	0	0	0.528	0	0
(A+P)+M+T	E	433.476	0.001	0.003	0.507	0	0	0	0	0	0	0.167	0.541	0	0	0.45	0	0
	N	422.47	0	0.001	0.056	0	0	0	0	0	0	0.044	0.282	0	0	0.009	0	0
	U	424.46	0	0.001	0.431	0	0	0	0	0	0	0.165	0.324	0	0	0.418	0	0
A+P+M+T	E	435.474	0.002	0.004	0.52	0	0	0	0	0	0	0.181	0.566	0	0	0.489	0	0
	N	424.468	0	0.001	0.082	0	0	0	0	0	0	0.05	0.291	0	0	0.019	0	0
	U	426.468	0	0.001	0.081	0	0	0	0	0	0	0.05	0.29	0	0	0.019	0	0
Time (5)	E	479.614	1	1	0.771	1	1	0.996	1	1	1	0.825	0.273	1	1	0.546	1	1
	N	501.382	1	1	0.981	1	0.998	1	0.981	1	1	0.968	0.318	1	0.999	0.957	1	1
	U	437.066	0	0	0.212	0	0	0	0	0	0	0.152	0.919	0	0	0.371	0	0
Time (21)	E	500.576	1	1	0.819	1	1	1	1	1	1	0.862	0.525	1	1	0.625	1	0.691
	N	521.298	1	1	0.997	1	1	1	1	1	1	0.986	0.53	1	1	0.963	1	0.706
	U	459.458	0.999	0.999	0.825	0.996	0.995	0.992	0.994	0.995	0.998	0.849	0.778	0.002	0	0.356	0.002	0.302

			P+M+T	A	A	P+M+T	P+T	P+M	P+T	P+M	P+T	M+T	Time (5)	M+T	A+M	P	M+T	P	M
		AIC	465.774	467.698	469.148	470.602	471.998	473.81	473.81	475.142	478.162	478.5	479.614	480.062	480.52	481.87	481.956	483.42	484.42
			U	E	U	E	N	E	U	U	E	N	E	U	N	E	E	U	E
E, N, U	E	494.778	0.981	1	0.516	1	0.997	1	0.754	0.457	0.997	0.997	0.989	0.615	1	1	1	0.586	1
	N	516.904	0.998	1	0.945	1	0.931	1	0.977	0.964	0.996	0.996	0.988	0.994	0.996	1	0.996	0.993	0.994
	U	496.646	0.981	0.998	0.51	1	0.931	1	0.739	0.458	0.996	0.996	0.986	0.61	0.998	1	0.992	0.483	0.994
A	E	467.698	0.589	0.94	0.733	0	0	0	0.466	0.663	0	0	0	0.535	0	0	0	0.89	0
	N	487.876	0.984	0.842	0.236	1	0.931	1	0.753	0.469	0.998	0.996	0.991	0.612	0.987	1	0.998	0.585	0.997
	U	469.148	1	0.005	0.438	0	0	0	0.041	0.101	0	0	0	0.05	0	0	0	0.023	0
P	E	481.87	0.534	0.998	0.37	1	0.997	1	0.456	0.265	0.68	0.997	0.989	0.425	1	0.07	0	0.928	0.001
	N	501.54	0.98	1	0.515	0.996	0.819	0.999	0.723	0.34	0.922	0.995	0.988	0.617	0.997	0.968	0.996	0.28	0.992
	U	483.42	0.998	0.998	0.919	0.994	0.837	0.991	0.911	0.957	0.859	0.995	0.989	0.686	1	0.797	0.993	0.29	0.009
M	E	484.42	0.559	1	0.371	0.981	0.996	0.973	0.514	0.288	0.991	0.996	0.986	0.393	1	1	0.634	0.086	1
	N	504.984	0.983	1	0.514	0.991	0.929	0.99	0.753	0.384	0.996	0.78	0.991	0.589	0.849	1	0.888	0.591	0.823
	U	486.418	0.995	0.999	0.872	0.985	0.932	0.974	0.904	0.892	0.991	0.791	0.985	0.893	0.854	0.999	0.83	0.906	0.822
T	E	491.866	0.558	1	0.372	1	0.997	1	0.481	0.32	0.99	0.997	0.994	0.391	1	1	1	0.085	1
	N	489.258	0.977	1	0.522	1	0.862	1	0.731	0.467	0.994	0.992	0.989	0.598	0.998	1	0.994	0.598	0.997
	U	490.698	1	0.999	0.954	1	0.916	1	0.971	0.962	0.984	0.996	0.987	0.599	1	1	0.992	0.997	0.992
A+T	E	463.184	0.427	0.001	0.714	0	0	0	0.486	0.648	0	0	0	0.558	0	0	0	0.876	0
	N	456.612	0	0.001	0.603	0	0	0	0.23	0.439	0	0	0	0.352	0	0	0	0.326	0
	U	458.472	0	0.002	0.009	0	0	0	0.034	0.1	0	0	0	0.054	0	0	0	0.015	0
P+T	E	478.162	0.487	0.999	0.372	1	0.977	1	0.416	0.271	0.854	0.001	0.004	0.602	0	0.006	0	0.921	0
	N	471.998	0.997	1	0.528	0.985	0.57	0	0.338	0.607	0.006	0	0.001	0.378	0	0.001	0	0.44	0
	U	473.81	0.989	0.999	0.925	0.975	0.945	0.995	0.758	0.013	0.017	0	0.002	0.017	0	0.003	0	0.004	0
M+T	E	481.956	0.519	1	0.373	0.979	0.996	0.988	0.482	0.288	0.99	0.981	0.99	0.358	1	1	0.954	0.91	0.016
	N	478.5	0.99	1	0.518	0.989	0.87	0.994	0.731	0.393	0.994	0.706	0.009	0.469	0.001	0	0.033	0.369	0.008
	U	480.062	0.988	0.999	0.869	0.981	0.966	0.983	0.873	0.891	0.982	0.964	0.99	0.976	0.001	0	0.072	0.091	0.012
A+M	E	461.504	0.43	0.007	0.712	0	0	0	0.45	0.676	0	0	0	0.546	0	0	0	0.876	0
	N	480.52	0.991	0.897	0.186	0.994	0.932	0.992	0.748	0.39	0.997	0.786	0.992	0.594	0.916	0	0.048	0.39	0.023
	U	462.758	0.001	0.012	0.153	0	0	0	0.11	0.174	0	0	0	0.105	0	0	0	0.104	0
P+M	E	473.81	0.512	0.999	0.37	0.673	0.998	0.994	0.542	0.753	0.01	0	0.002	0.584	0	0.003	0	0.916	0.001
	N	492.24	0.987	1	0.516	0.917	0.832	0.842	0.727	0.128	0.947	0.788	0.99	0.593	0.856	0.967	0.907	0.297	0.894
	U	475.142	0.961	0.999	0.854	0.846	0.955	0.803	0.858	0.578	0.032	0.006	0.005	0.05	0.001	0.011	0.006	0.186	0.003
A+P	E	444.428	0.388	0	0.655	0	0	0	0.442	0.644	0	0	0	0.454	0	0	0	0.866	0
	N	462.46	0	0.005	0.645	0	0	0	0.244	0.541	0	0	0	0.348	0	0	0	0.377	0
	U	443.742	0	0	0.03	0	0	0	0.031	0.1	0	0	0	0.041	0	0	0	0.091	0
(A+P)+T	E	436.002	0.378	0	0.589	0	0	0	0.431	0.622	0	0	0	0.471	0	0	0	0.827	0
	N	425.146	0	0	0.382	0	0	0	0.183	0.407	0	0	0	0.299	0	0	0	0.291	0
	U	427.132	0	0	0.005	0	0	0	0.018	0.085	0	0	0	0.002	0	0	0	0.052	0
A+P+T	E	438	0	0	0.482	0	0	0	0.214	0.426	0	0	0	0.314	0	0	0	0.325	0
	N	427.142	0	0	0.019	0	0	0	0.031	0.096	0	0	0	0.05	0	0	0	0.063	0
	U	429.13	0	0	0.426	0	0	0	0.214	0.4	0	0	0	0.312	0	0	0	0.352	0
A+M+T	E	457.528	0	0	0.604	0	0	0	0.232	0.489	0	0	0	0.373	0	0	0	0.323	0
	N	450.542	0	0	0.127	0	0	0	0.108	0.158	0	0	0	0.097	0	0	0	0.101	0
	U	452.424	0	0	0.565	0	0	0	0.226	0.459	0	0	0	0.374	0	0	0	0.326	0
P+M+T	E	470.602	0.966	0.999	0.521	0.974	0.011	0.006	0.311	0.691	0.006	0.001	0	0.401	0	0	0	0.418	0
	N	463.93	0.478	0	0.089	0.011	0.002	0	0.122	0.015	0.001	0	0	0.044	0	0	0	0.143	0
	U	465.774	0.71	0	0.46	0.03	0.003	0.003	0.316	0.621	0.001	0	0	0.399	0	0	0	0.732	0
A+P+M	E	441.29	0	0	0.518	0	0	0	0.208	0.466	0	0	0	0.325	0	0	0	0.329	0
	N	458.42	0.001	0.007	0.19	0.001	0	0	0.129	0.172	0	0	0	0.117	0	0	0	0.218	0
	U	440.472	0	0	0.51	0	0	0	0.215	0.462	0	0	0	0.325	0	0	0	0.376	0
(A+P)+M+T	E	433.476	0	0	0.403	0	0	0	0.19	0.424	0	0	0	0.319	0	0	0	0.301	0
	N	422.47	0	0	0.061	0	0	0	0.068	0.073	0	0	0	0.011	0	0	0	0.13	0
	U	424.46	0	0	0.374	0	0	0	0.201	0.396	0	0	0	0.314	0	0	0	0.301	0
A+P+M+T	E	435.474	0	0	0.452	0	0	0	0.211	0.443	0	0	0	0.32	0	0	0	0.321	0
	N	424.468	0	0	0.081	0	0	0	0.087	0.139	0	0	0	0.079	0	0	0	0.153	0
	U	426.468	0	0	0.081	0	0	0	0.087	0.139	0	0	0	0.08	0	0	0	0.154	0
Time (5)	E	479.614	0.999	1	0.531	1	1	1	0.76	0.486	0.997	1	0.75	0.379	0	0	0.001	0.363	0
	N	501.382	1	1	0.924	1	0.989	1	0.931	0.937	0.997	1	0.761	0.985	0.999	1	0.999	0.936	0.996
	U	437.066	0	0	0.35	0	0	0	0.193	0.393	0	0	0	0.296	0	0	0	0.297	0
Time (21)	E	500.576	0.718	0.775	0.633	0.778	0.999	1	0.797	0.578	0.999	1	1	0.676	1	1	0.738	0.674	1
	N	521.298	0.799	0.896	0.96	0.892	1	1	0.957	0.965	1	1	1	0.991	1	1	0.875	0.092	1
	U	459.458	0.264	0.186	0.336	0.189	0.002	0.001	0.191	0.381	0	0	0.002	0.292	0.002	0	0.181	0.297	0.001

			M	A	T	T	T	P+M	E	U	Time (21)	Time (5)	P	M	N	Time (21)
		AIC	486.418	487.876	489.258	490.698	491.866	492.24	494.778	496.646	500.576	501.382	501.54	504.984	516.904	521.298
			U	N	N	U	E	N	E	U	E	N	N	N	N	N
E, N, U	E	494.778	0.268	0.997	0.999	0.569	0.654	0.997	0.87	0.222	0.751	0	0	0	0	0.004
	N	516.904	0.856	0.84	0.797	0.946	0.923	0.994	0.841	0.851	0.584	0.926	0.829	0.998	0.546	0.783
	U	496.646	0.273	0.844	0.815	0.561	0.871	0.997	0.834	0.974	0.787	0.016	0	0	0	0.007
A	E	467.698	0.77	0	0	0.613	0	0	0	0.544	0	0	0	0	0	0
	N	487.876	0.273	0.794	0.003	0.42	0.008	0.001	0.007	0.003	0.191	0	0	0	0	0
	U	469.148	0.115	0	0	0.101	0	0	0	0	0	0	0	0	0	0
P	E	481.87	0.8	0	0	0.659	0	0	0	0.594	0.114	0	0	0	0	0
	N	501.54	0.274	0.84	0.809	0.567	0.941	0.988	0.901	0.545	0.596	0.943	0.162	0	0	0.09
	U	483.42	0.036	0	0.002	0.047	0.001	0	0	0	0.144	0	0	0	0	0
M	E	484.42	0.823	0	0	0.671	0.002	0	0.001	0.603	0.195	0	0	0	0	0
	N	504.984	0.063	0.839	0.806	0.57	0.94	0.826	0.901	0.565	0.595	0.941	0.827	0.592	0	0.224
	U	486.418	0.436	0	0.009	0.141	0.009	0	0.006	0	0.256	0	0	0	0	0
T	E	491.866	0.197	0.999	0.981	0.273	0.898	0.003	0.007	0.633	0.552	0	0	0	0	0
	N	489.258	0.276	0.844	0.504	0.503	0.04	0.001	0.01	0.004	0.225	0.002	0	0	0	0
	U	490.698	0.853	0.999	0.962	0.898	0.095	0.001	0.018	0.002	0.457	0	0	0	0	0
A+T	E	463.184	0.762	0	0	0.64	0	0	0	0.516	0	0	0	0	0	0
	N	456.612	0.615	0	0	0.363	0	0	0	0	0	0	0	0	0	0
	U	458.472	0.117	0	0	0.104	0	0	0	0	0	0	0	0	0	0
P+T	E	478.162	0.794	0	0	0.683	0	0	0	0.576	0.031	0	0	0	0	0
	N	471.998	0.665	0	0	0.392	0	0	0	0	0.005	0	0	0	0	0
	U	473.81	0.031	0	0	0.046	0	0	0	0	0.006	0	0	0	0	0
M+T	E	481.956	0.817	0	0	0.696	0.001	0	0.001	0.594	0.076	0	0	0	0	0
	N	478.5	0.738	0	0	0.415	0.001	0	0	0	0.025	0	0	0	0	0
	U	480.062	0.031	0	0	0.186	0	0	0	0	0.033	0	0	0	0	0
A+M	E	461.504	0.782	0	0	0.592	0	0	0	0.512	0	0	0	0	0	0
	N	480.52	0.748	0.004	0.001	0.403	0.001	0	0.001	0	0.038	0	0	0	0	0
	U	462.758	0.139	0	0	0.187	0	0	0	0	0	0	0	0	0	0
P+M	E	473.81	0.804	0	0	0.632	0	0	0	0.566	0.004	0	0	0	0	0
	N	492.24	0.122	0.955	0.921	0.57	0.961	0.622	0.027	0.032	0.274	0.004	0.003	0	0	0.004
	U	475.142	0.086	0	0	0.129	0	0	0	0	0.008	0	0	0	0	0
A+P	E	444.428	0.724	0	0	0.53	0	0	0	0.446	0	0	0	0	0	0
	N	462.46	0.64	0	0	0.349	0	0	0	0	0	0	0	0	0	0
	U	443.742	0.122	0	0	0.081	0	0	0	0	0	0	0	0	0	0
(A+P)+T	E	436.002	0.711	0	0	0.547	0	0	0	0.423	0	0	0	0	0	0
	N	425.146	0.526	0	0	0.302	0	0	0	0	0	0	0	0	0	0
	U	427.132	0.026	0	0	0.019	0	0	0	0	0	0	0	0	0	0
A+P+T	E	438	0.556	0	0	0.328	0	0	0	0	0	0	0	0	0	0
	N	427.142	0.115	0	0	0.083	0	0	0	0	0	0	0	0	0	0
	U	429.13	0.531	0	0	0.327	0	0	0	0	0	0	0	0	0	0
A+M+T	E	457.528	0.652	0	0	0.364	0	0	0	0	0	0	0	0	0	0
	N	450.542	0.136	0	0	0.19	0	0	0	0	0	0	0	0	0	0
	U	452.424	0.631	0	0	0.361	0	0	0	0	0	0	0	0	0	0
P+M+T	E	470.602	0.698	0	0	0.388	0	0	0	0	0.001	0	0	0	0	0
	N	463.93	0.052	0	0	0.149	0	0	0	0	0	0	0	0	0	0
	U	465.774	0.673	0	0	0.392	0	0	0	0	0	0	0	0	0	0
A+P+M	E	441.29	0.598	0	0	0.32	0	0	0	0	0	0	0	0	0	0
	N	458.42	0.151	0	0	0.191	0	0	0	0	0	0	0	0	0	0
	U	440.472	0.592	0	0	0.32	0	0	0	0	0	0	0	0	0	0
(A+P)+M+T	E	433.476	0.577	0	0	0.32	0	0	0	0	0	0	0	0	0	0
	N	422.47	0.043	0	0	0.104	0	0	0	0	0	0	0	0	0	0
	U	424.46	0.537	0	0	0.317	0	0	0	0	0	0	0	0	0	0
A+P+M+T	E	435.474	0.581	0	0	0.326	0	0	0	0	0	0	0	0	0	0
	N	424.468	0.125	0	0	0.172	0	0	0	0	0	0	0	0	0	0
	U	426.468	0.125	0	0	0.172	0	0	0	0	0	0	0	0	0	0
Time (5)	E	479.614	0.685	0	0	0.386	0	0	0	0	0.807	0	0	0	0	0
	N	501.382	0.802	0.987	0.98	0.847	0.982	0.999	0.96	0.97	0.447	0.424	0.012	0	0	0.878
	U	437.066	0.549	0	0	0.308	0	0	0	0	0	0	0	0	0	0
Time (21)	E	500.576	0.374	0.999	1	0.652	1	1	1	1	0.726	0	0	0	0	0
	N	521.298	0.862	1	1	0.908	1	1	1	1	0.737	1	1	1	0.999	0.558
	U	459.458	0.527	0.001	0	0.301	0	0.001	0	0	0.001	0.001	0	0	0	0
